# *Caulobacter crescentus* CdnL is a non-essential RNA polymerase-binding protein whose depletion impairs normal growth and rRNA transcription

**DOI:** 10.1038/srep43240

**Published:** 2017-02-24

**Authors:** Aránzazu Gallego-García, Antonio A. Iniesta, Diego González, Justine Collier, S. Padmanabhan, Montserrat Elías-Arnanz

**Affiliations:** 1 Departamento de Genética y Microbiología, Área de Genética (Unidad Asociada al IQFR-CSIC), Facultad de Biología, Universidad de Murcia, 30100 Murcia, Spain; 2 Department of Fundamental Microbiology, Faculty of Biology and Medicine, University of Lausanne, Quartier UNIL/Sorge, Lausanne, CH1015, Switzerland; 3 Instituto de Química Física ‘Rocasolano’, Consejo Superior de Investigaciones Científicas (IQFR-CSIC), Serrano 119, 28006 Madrid, Spain

## Abstract

CdnL is an essential RNA polymerase (RNAP)-binding activator of rRNA transcription in mycobacteria and myxobacteria but reportedly not in *Bacillus*. Whether its function and mode of action are conserved in other bacteria thus remains unclear. Because virtually all alphaproteobacteria have a CdnL homolog and none of these have been characterized, we studied the homolog (CdnL_Cc_) of the model alphaproteobacterium *Caulobacter crescentus*. We show that CdnL_Cc_ is not essential for viability but that its absence or depletion causes slow growth and cell filamentation. CdnL_Cc_ is degraded *in vivo* in a manner dependent on its C-terminus, yet excess CdnL_Cc_ resulting from its stabilization did not adversely affect growth. We find that CdnL_Cc_ interacts with itself and with the RNAP β subunit, and localizes to at least one rRNA promoter *in vivo*, whose activity diminishes upon depletion of CdnL_Cc_. Interestingly, cells expressing CdnL_Cc_ mutants unable to interact with the RNAP were cold-sensitive, suggesting that CdnL_Cc_ interaction with RNAP is especially required at lower than standard growth temperatures in *C. crescentus*. Our study indicates that despite limited sequence similarities and regulatory differences compared to its myco/myxobacterial homologs, CdnL_Cc_ may share similar biological functions, since it affects rRNA synthesis, probably by stabilizing open promoter-RNAP complexes.

Bacteria adapt to changing conditions by controlling gene expression, and transcription initiation is the most frequently regulated step. Bacterial transcription begins when the RNA polymerase (RNAP) holoenzyme, consisting of the core (α_2_ββ′ω) and a specific dissociable σ factor, recognizes a target promoter to form a closed complex (RP_c_), which undergoes a series of conformational changes leading to open complex (RP_o_) formation and RNA synthesis from the DNA template^1^,^2^,^3^. Defined promoter elements and the choice of a given σ factor are fundamental determinants of target specificity. Nonetheless, numerous accessory factors often play key roles in modulating, positively or negatively, the various steps of transcription initiation.

An emerging class of bacterial RNAP-interacting transcriptional factors are members of the CarD-CdnL family. The global transcriptional regulator CarD, composed of an N-terminal RNAP-interacting domain and a DNA-binding domain resembling eukaryotic HMGA proteins, is found only in myxobacteria, where it is dispensable for cell viability, and has been linked to the action of the alternative extracytoplasmic function (ECF) σ factors^4^,^5^,^6^,^7^. By contrast, CdnL (for CarD N-terminal like) is a standalone version of the N-terminal domain of CarD, whose homologs (sometimes referred to as CarD) occur in several bacterial taxonomical groups^5^,^8^,^9^,^10^. CdnL from *M. xanthus* (CdnL_Mx_) and its *Mycobacterium tuberculosis* homolog (CdnL_Mt_) are essential for cell viability^9^,^10^, as may also be the case for the homolog in the spirochaete *Borrelia burgdorferi*^11^. On the other hand, CdnL is reportedly not essential in *Bacillus subtilis*^12^,^13^ or in *Bacillus cereus*^14^, suggesting that its function is not always conserved. In fact, many members of the phylum Firmicutes, to which *B. subtilis* and *B. cereus* belong, lack a CdnL homolog.

The critical function of CdnL is likely related to its role in promoting gene expression by stabilizing RP_o_ formation at promoters, such as those for rRNA, that depend on the primary σ factor^15^,^16^,^17^,^18^,^19^. Like the CarD N-terminal domain, CdnL_Mx_ interacts with the RNAP β subunit (RNAPβ) but, while this interaction is dispensable for CarD, it is crucial for CdnL_Mx_^16^,^20^. The CdnL-RNAPβ interaction has also been demonstrated for CdnL_Mt_^21^, CdnL_Tt_ in *Thermus thermophilus*^10^,^22^, CdnL_Bb_ in *Bdellovibrio bacteriovorus* (a deltaproteobacterium like *M. xanthus*), CdnL_Cg_ in *Corynebacterium glutamicum* and CdnL_Sc_ in *Streptomyces coelicolor*, both actinobacteria like *M. tuberculosis*^16^. The *B. subtilis* homolog CdnL_Bs_ (also named YdeB), however, does not appear to interact with RNAPβ^13^.

To establish if CdnL has conserved functions, and to gain insights into structure-function relationships in this large family of proteins, it is imperative to characterize CdnL from diverse classes of bacteria. *Caulobacter crescentus*, a Gram-negative oligotrophic fresh water alphaproteobacterium, is an important model system for studies on bacterial cell cycle, division and differentiation^23^. The alphaproteobacteria class includes members with very varied lifestyles and, although most of them have a CdnL homolog, none had been specifically studied. This prompted us to characterize the homolog in *C. crescentus,* CdnL_Cc_ (26–34% identical to homologs studied in other bacterial species; Supplementary Table S1, Supplementary Fig. S1). Our findings indicate that CdnL_Cc_ is required for normal growth in *C. crescentus* but is not essential for viability, in contrast to CdnL_Mt_ and CdnL_Mx_. Our data show that CdnL_Cc_ is degraded *in vivo* and that this depends on its C-terminal AA motif, although stabilizing CdnL_Cc_ had no apparent effect on growth. We also show that CdnL_Cc_ binds to RNAPβ and localizes to at least one rRNA promoter *in vivo*, whose activity diminishes on limiting intracellular CdnL_Cc_ levels. Given these parallels with CdnL_Mx_, CdnL_Mt_, and CdnL_Tt_, we propose that CdnL_Cc_ may also stabilize open rRNA promoter-RNAP complexes. Interestingly, we find that missense mutations of conserved residues of CdnL_Cc_ that impair the interaction with RNAPβ lead to a cold-sensitive *in vivo* phenotype, suggesting that CdnL_Cc_ interaction with RNAP is functionally more important at lower temperatures in *C. crescentus*. Our results extend the requirement of CdnL for normal cell growth and its possible functional roles in alphaproteobacteria and contribute to elucidating structure-function relationships underlying its mode of action.

## Results

### CdnL is widespread and highly conserved in alphaproteobacteria

A CdnL homolog was found in all representative alphaproteobacteria, except for the early diverging genus *Magnetococcus*, a clade of Rickettsiales comprising the genera *Neorickettsia, Wolbachia, Ehrlichia* and *Anaplasma*, and a single Rhodospirillales (*Thalassobaculum_L2*). A phylogenetic tree of these proteins (Supplementary Fig. S2) is globally consistent with the accepted phylogeny of alphaproteobacteria^24^, suggesting that CdnL has mostly been transmitted vertically along the phylogeny without long-range lateral gene transfer. Given that other classes of proteobacteria have a CdnL homolog, the most parsimonious hypothesis is that the common ancestor of alphaproteobacteria had a CdnL homolog that was lost along one of the main branches in the Rickettsiales group and, independently, in *Magnetococcus*. The presence of CdnL in most alphaproteobacteria, with high overall amino acid sequence conservation (>50% identity and >90% coverage relative to CdnL_Cc_) suggests strong purifying selection and an important cellular function. Studies with CdnL_Cc_ could thus help understand its role not only in *C. crescentus* but also in other alphaproteobacteria.

### CdnL_Cc_ is not essential for viability but is required for normal cell growth

A recent global transcriptional start site (TSS) mapping study in *C. crescentus*^25^ listed a single TSS for *cdnL*_Cc_ and assigned a putative −35 to −10 promoter segment (TTCATAG-x_12_-GCTATTGT, where x is A, T, C, or G) similar to the consensus for promoters dependent on σ^73^, the primary σ factor in *C. crescentus* (5′-TTGaCg(c/g)-x_11-14_-GCtAxA(a/t)C-3′^26^,^27^). We found that a reporter *lacZ* fusion to the 352-bp intergenic region upstream of *cdnL*_Cc_, which includes the predicted σ^73^-dependent promoter, showed high and increasing reporter β-galactosidase activity during exponential growth that leveled off in stationary phase (Supplementary Fig. S3). Interestingly and notably different from *cdnL*_Mx_ and *cdnL*_Mt_, the mapped *cdnL*_Cc_ TSS suggests a long (233 nt) 5′untranslated region (5′-UTR), comparable to the 266-nt one reported for the alphaproteobacterium *Sinorhizobium meliloti* in another global TSS study^28^.

**Figure 1:**
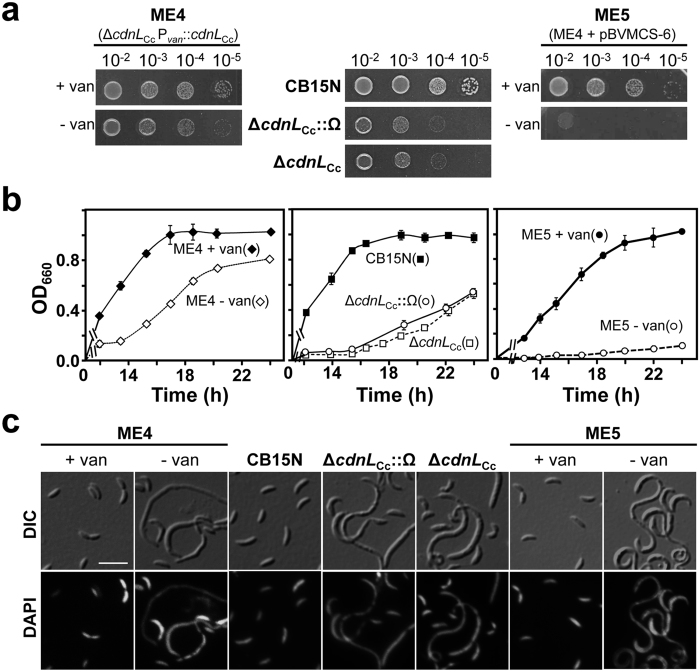
Growth and cell morphology upon CdnL_Cc_ depletion. (**a**) Growth of the indicated *C. crescentus* strains. Liquid cultures (OD_660_ ∼ 0.5) were serially diluted, spotted (8 μl) on PYE plates with (+van) or without (−van) 0.5 mM vanillate, and incubated for 2 days at 30 °C. (**b**) Growth curves for the strains indicated (with symbols in parentheses). Freshly plated cells were innoculated into 10 ml of PYE (with 0.5 mM vanillate for ME4 and ME5) and grown at 30 °C to OD_660_ ∼ 0.8. 50 μl were aliquoted (for ME4 and ME5, after washing three times with PYE to remove the vanillate) into 10 ml of fresh PYE (for ME4 and ME5, one with 0.5 mM vanillate and one without). Growth was monitored at the indicated times, and the average and error of three independent measurements is shown. (**c**) Cellular morphology of cells from (**b**). Samples (concentrated ten-fold in the case of ME5 cultivated in the absence of vanillate) of each culture in (**b**) after ∼20 h of growth were DAPI-stained and examined by DIC (differential interference contrast; top panels) and fluorescence microscopy (bottom panels), as described in Methods. Scale bar: 5 μm.

A genome-wide transposon insertion analysis suggested that *cdnL*_Cc_ is essential, or at least has a strong fitness impact, when *C. crescentus* is cultivated in rich medium^29^. Similar studies in other alphaproteobacteria have also listed *cdnL* as essential in *Brevundimonas subvibrioides* (Caulobacterales, like *C. crescentus*)^30^ and *Rhodopseudomonas palustris* (Rhizobiales)^31^, but it was unclear in *Agrobacterium tumefaciens*^30^ and in *Rhizobium leguminosarum*^32^ (both Rhizobiales). We therefore probed the functional importance of *cdnL*_Cc_ by first attempting to generate a markerless, in-frame *cdnL*_Cc_ deletion (Δ*cdnL*_Cc_) with a two-step allele exchange strategy (see Supplementary Methods). Inability to obtain haploid cells with the Δ*cdnL*_Cc_ allele (none out of ∼75 colonies analyzed that grew after two days had the Δ*cdnL*_Cc_ allele) hinted that *cdnL*_Cc_ may be essential for viability. This was further supported by the ability to delete *cdnL*_Cc_ when a second functional copy, expressed under the control of the vanillate-inducible P_*van*_ promoter, was supplied at a heterologous chromosomal site: approximately a third of the colonies analyzed after the normal two-day growth now had the Δ*cdnL*_Cc_ allele (strain ME4). However, ME4 grew without vanillate in the medium, albeit slower than in the presence of the inducer (doubling time ∼140 min versus ∼90 min; Fig. 1a,b), suggesting leaky P_*van*_ expression in the absence of vanillate or that *cdnL*_Cc_ is not essential. To resolve this issue, we attempted to replace *cdnL*_Cc_ by a Δ*cdnL*_Cc_::Ω allele (conferring spectinomycin/streptomycin resistance) using the two-step allele exchange strategy in the presence of a complementing plasmid, followed by generalized transduction of the Spec^R^/Strep^R^ Ω cassette into the wild-type strain (see Supplementary Methods). A significant number of Spec^R^/Strep^R^ colonies (30–50) grew ∼4–5 days after transduction, which were confirmed to have the Δ*cdnL*_Cc_::Ω allele (strain JC784), suggesting that CdnL_Cc_ is in fact dispensable but its absence slows down growth (Fig. 1a,b). We therefore repeated our attempt to obtain a markerless Δ*cdnL*_Cc_ strain, plating increasing dilutions to enable detection of slow-growing colonies. Such colonies emerged after ∼4–5 days, and all of them had the Δ*cdnL*_Cc_ allele (strain ME50). It is unlikely that these Δ*cdnL*_Cc_ colonies appeared as a consequence of supressor mutations, since they were isolated at a frequency comparable to that observed when a complementing *cdnL*_Cc_ copy was present. Furthermore, both Δ*cdnL*_Cc_ strains (JC784 and ME50), even though generated independently, exhibited the same slow-growth behavior in rich medium (Fig. 1a), with similar doubling times (∼230 min; Fig. 1b), which were higher than for the wild type (∼90 min) or for ME4 in the absence of vanillate (∼140 min). Many JC784 and ME50 cells, and ME4 cells grown in the absence of vanillate, were filamentous indicating cell division defects, and the DNA visualized by DAPI fluorescence was often unevenly distributed (Fig. 1c).

The faster growth rate of ME4 (with no vanillate) relative to JC784 or ME50, suggests leaky P_*van*_-*cdnL*_Cc_ expression. To achieve tighter repression, we introduced a high-copy number plasmid expressing *vanR*, pBVMCS-6^27^, into ME4. Growth of the resulting strain (ME5; Fig. 1a–c) in the absence of vanillate was more severely affected than that of ME4 but, curiously, even more so than that of the two Δ*cdnL*_Cc_ strains (JC784 or ME50). This was also observed when a high-copy number plasmid bearing *vanR* with a different antibiotic resistance marker (pBVMCS-2) was used, or even when such plasmids lacked *vanR* (Supplementary Fig. S4)^27^. Thus, CdnL_Cc_ depletion appears to undermine the ability of cells to cope with the fitness burden due to the presence of these plasmids. The possibility of turning CdnL_Cc_ expression on or off in ME5, together with its more severe growth phenotype upon depleting CdnL_Cc_, led us to use this strain for assessing functionality of CdnL_Cc_ variants in *C. crescentus* (below).

In sum, CdnL_Cc_ is not essential under standard growth conditions on rich medium but its absence or its depletion causes growth and morphological defects, and compromises cell fitness and ability to deal with stresses such as maintaining high-copy number plasmids in *C. crescentus*.

### CdnL_Cc_ is degraded *in vivo* in a manner dependent on its C-terminus, but its stabilization does not impair cell growth

**Figure 2:**
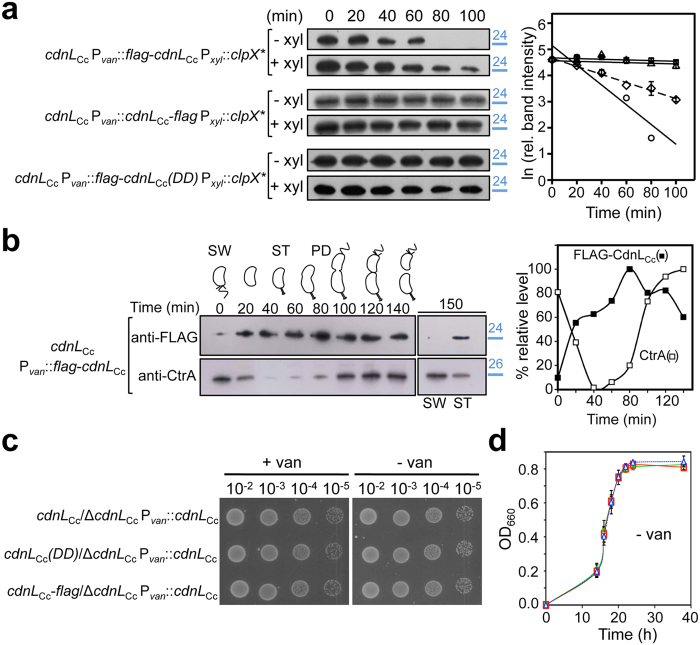
Analysis of CdnL_Cc_ protein stability *in vivo*. (**a**) Stability of FLAG-CdnL_Cc,_ CdnL_Cc_-FLAG and FLAG-CdnL_Cc_(DD) *in vivo. C. crescentus* strains ME27, ME28 and ME29 were grown in PYE with vanillate, and subjected to a previously described protocol^35^ (see Methods) prior to immunoblot analysis (−xyl: no xylose; +xyl: 0.3% xylose to induce clpX* expression). Total protein from 1 ml aliquots withdrawn every 20 min was detected in immunoblots using anti-FLAG antibodies (left). On the right is a semi-log plot of the relative band intensities (mean of three independent experiments) versus time for FLAG-CdnL_Cc_ (−xyl, circles; +xyl, diamonds), CdnL_Cc_-FLAG (−xyl, triangles) and FLAG-CdnL_Cc_(DD) (−xyl, squares). Slopes of the linear fits shown yield the decay rate constants used to estimate half-lives. (**b**) FLAG-CdnL_Cc_ levels during the cell cycle. Swarmer cells (SW) from strain ME24 grown in M2G with vanillate were isolated and used for synchronized cell cycle progression (∼150 min doubling time). Total protein from 1 ml aliquots taken every 20 min was subjected to immunoblot analysis using anti-FLAG antibodies. The control CtrA was probed on a separate blot (since its gel mobility is close to that of FLAG-CdnL_Cc_) using anti-CtrA antibodies^38^ and equivalent samples from the same experiment processed in parallel. Samples at 150 min correspond to SW and ST (with a small proportion of PD) cells isolated from the culture remaining at the end of this assay. A plot of band intensities (% of the maximum value) versus time is shown (right). In (**a**,**b**), positions of molecular size markers (kDa) are shown in blue to the right of cropped immunoblots. Note that FLAG-tagged CdnL_Cc_ (∼19.5 kDa calculated Mw) migrates slower than expected. (**c**) Growth of *C. crescentus* strains (ME41, ME39, ME8) expressing CdnL_Cc_, CdnL_Cc_(DD) or CdnL_Cc_-FLAG, respectively, at the endogenous site. Liquid cultures (OD_660_ ∼ 0.5) were serially diluted, spotted (8 μl) on PYE plates with or without vanillate, and incubated for 2 days at 30 °C. (**d**) Growth curves of strains in (**c**) expressing CdnL_Cc_ (squares, red), CdnL_Cc_-FLAG (triangles, blue) or CdnL_Cc_(DD) (circles, green) in PYE without vanillate at 30 °C.

CdnL_Cc_ has a C-terminal AA motif (Supplementary Fig. S1), a hallmark of many substrates of the energy-dependent ClpXP protease, essential in *C. crescentus*, where it degrades several proteins implicated in replication, cell cycle or development^33^,^34^. We therefore examined the stability of CdnL_Cc_ *in vivo*. For this, CdnL_Cc_ was fused to a FLAG epitope tag, to enable detection by immunoblot analysis. Since the tag can mask potential protease recognition, both N- and C-terminally tagged versions were tested. We could generate a normally growing strain expressing the C-terminally FLAG-tagged CdnL_Cc_ (CdnL_Cc_-FLAG) as the sole CdnL_Cc_ copy, but not one with only the N-terminally FLAG-tagged CdnL_Cc_ (FLAG-CdnL_Cc_), suggesting that an N-terminal tag impairs CdnL_Cc_ function. We therefore used strains expressing *cdnL*_Cc_ from its native locus (to ensure normal growth) and the given FLAG-tagged CdnL_Cc_ from a tightly controlled vanillate-inducible P_*van*_ promoter. To test if CdnL_Cc_ is degraded in a ClpX-dependent manner *in vivo*, we introduced, as reported previously^35^, a plasmid (pM088) allowing xylose-inducible expression of the dominant-negative ClpX* mutant chaperone (which is altered in its ATP-binding site and produces a catalytically dead ClpX form)^36^. Immunoblot analysis of FLAG-CdnL_Cc_ degradation after turning off its expression (Fig. 2a) revealed that its stability was enhanced about two-fold when ClpX* expression was induced (estimated half-life of 45 ± 3 min, versus 18 ± 4 min when ClpX* was not induced). Moreover, CdnL_Cc_-FLAG and FLAG-CdnL_Cc_(DD), with the C-terminal AA motif masked or replaced by two aspartates, respectively, were considerably more stable whether or not ClpX* expression was induced (half-lives of ∼300–580 min *in vivo*; Fig. 2a). Taken together, these results indicate that the AA motif at the C-terminus of CdnL_Cc_ is an important determinant for its degradation *in vivo* and suggest that this might be dependent, at least in part, on ClpX.

*C. crescentus* undergoes asymmetric cell division to produce a non-replicative swarmer cell (SW) and a stalked cell (ST) that reinitiates the replicative cell cycle. Proper cell cycle progression depends on regulated proteolysis, often mediated by ClpXP^33^,^35^. The aberrant cell division phenotype caused by CdnL_Cc_ depletion and the observation that it is subject to proteolysis prompted us to examine the levels of FLAG-CdnL_Cc_ (expressed from P_*van*_) along the cell cycle. As a control for synchronization and cell cycle progression, we also monitored the changes in the levels of CtrA, a master cell-cycle regulator and known ClpXP substrate, which is stable in SW cells, then gets degraded in ST cells and again reappears in predivisional (PD) cells^34^,^37^,^38^. FLAG-CdnL_Cc_ increased from barely detectable levels in SW cells to peak at ∼80 min (Fig. 2b). Interestingly, the clearance of FLAG-CdnL_Cc_ from SW cells resembles that reported for the cell division protein FtsZ, a ClpXP and ClpAP substrate that, like CdnL_Cc_, is stabilized by tagging its C-terminus or by mutating it to a DD motif^35^.

The normal growth in the absence of vanillate of strain ME8, which expresses CdnL_Cc_-FLAG from the native promoter and also bears a copy of *cdnL*_Cc_ under P_*van*_ control, suggests that enhancing the stability of CdnL_Cc_ and its resulting accumulation *in vivo* are not toxic to the cells (Fig. 2c). We confirmed this further with cells expressing only untagged CdnL_Cc_(DD) that, like equivalent strains expressing only CdnL_Cc_ or CdnL_Cc_-FLAG, grew normally in the absence of vanillate on plates (Fig. 2c) or in liquid media (Fig. 2d). Thus, curiously, even though CdnL_Cc_ is targeted for degradation *in vivo*, preventing this degradation by mutating the AA motif to DD or masking it with a C-terminal tag does not appear detrimental to *C. crescentus* growth or viability.

### CdnL_Cc_ interacts with itself and with RNAPβ

Full-length CdnL_Cc_ is overall basic like CdnL_Tt_, and not acidic like CdnL_Mx_ or CdnL_Mt_ (Supplementary Table S1). High-resolution tertiary structures determined for CdnL_Tt_, CdnL_Mt_ and CdnL_Mx_ revealed a ∼70-residue N-terminal β-sheet module and a C-terminal α-helical domain comprising the rest of the protein^16^,^18^,^19^,^22^,^39^,^40^ that correspond well with sequence-based predictions of secondary structure (PSIPRED; http://bioinf.cs.ucl.ac.uk/psipred). Compared to its homologs, the putative CdnL_Cc_ N-terminal domain is 32–39% identical and is also acidic (only in CdnL_Tt_ it is basic), whereas its C-terminal domain, only 22–31% identical to the rest, is markedly basic, as in CdnL_Bs_ (Supplementary Table S1). Despite the low sequence identity and the divergent overall domain charge distribution, the predicted CdnL_Cc_ secondary structure (Supplementary Fig. S5) mirrors those in the high-resolution structures of CdnL_Tt_, CdnL_Mt_ and CdnL_Mx_.

**Figure 3:**
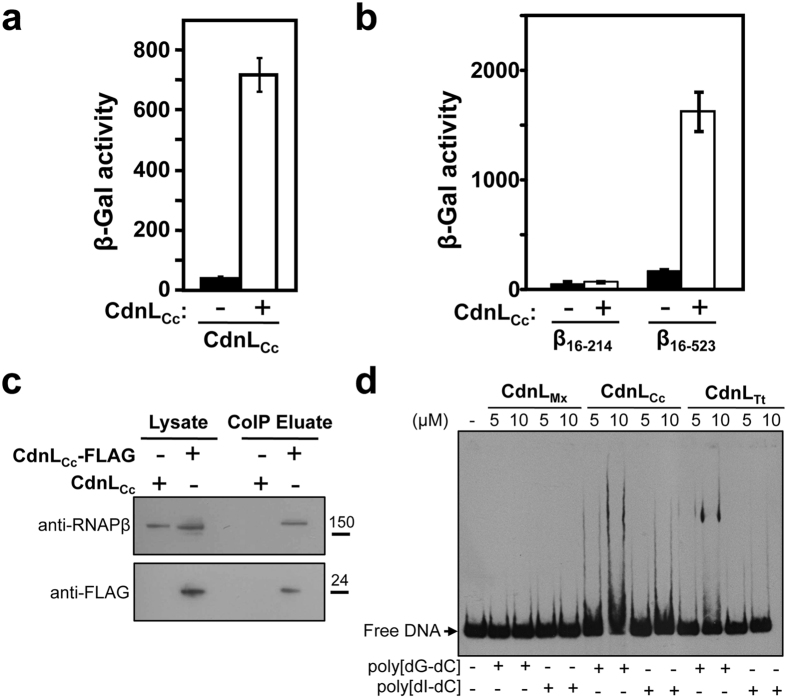
CdnL_Cc_ interacts with itself, with RNAP, and with DNA. (**a**) BACTH analysis of CdnL_Cc_ self-interaction in *E. coli* BTH101 transformed with plasmids pUT18-*cdnL*_Cc_ and pKT25-*cdnL*_Cc_. (**b**) BACTH analysis of the interaction between CdnL_Cc_ (in pKT25) and *C. crescentus* RNAP β subunit fragments β_16-214_ and β_16-523_ (in pUT18C). In (**a**,**b**), the negative control (-) was pKT25 without insert. (**c**) Western blot of immunoprecipitated CdnL_Cc_-FLAG probed for the presence of coprecipitating RNAPβ. Cells expressing CdnL_Cc_-FLAG (strain ME17: Δ*cdnL*_Cc_, P_*van*_::*cdnL*_Cc_-*flag, vanR*) or, as the negative control, untagged CdnL_Cc_ (strain ME5: Δ*cdnL*_Cc_, P_*van*_::*cdnL*_Cc_, *vanR*) were immunoprecipitated with anti-FLAG agarose and processed in parallel, as described in Methods. Equal amounts of sample were then resolved by SDS-PAGE for immunoblot analysis. Monoclonal anti-RNAP β antibodies were used to detect RNAPβ (top) that coimmunoprecipitated with CdnL_Cc_-FLAG, which was detected using anti-FLAG antibodies (bottom). Molecular size markers are shown to the right of the cropped immunoblots by the lines and corresponding values in kDa. (**d**) EMSA for the DNA binding of CdnL_Cc_, CdnL_Tt_, and CdnL_Mx_. Reactions were performed as described in Methods with a 350-bp P_*rrnA*_ DNA probe, and with 1 μg of poly[dG-dC] or poly[dI-dC] added as nonspecific competitor.

Interaction with cognate RNAPβ, a hallmark of most CdnL homologs studied thus far, was mapped to a solvent-exposed surface on the N-terminal β1 lobe of RNAPβ and to the ∼70-residue N-terminal module in CdnL^9^,^16^,^21^,^39^, which also mediates self-interactions^16^,^40^. Bacterial two-hybrid analysis (BACTH) confirmed that CdnL_Cc_ conserves the ability to self-interact (Fig. 3a). A ~120 residue fragment of RNAPβ corresponding to the β1a subdomain (where residues critical for interaction with other CdnL homologs are located) was sufficient to detect interaction between CdnL_Mx_ or CdnL_Tt_ and their respective RNAPβ in BACTH^9^,^10^. However, in similar assays with other CdnL homologs, a longer ∼500-residue fragment corresponding to the whole β1 domain (subdomains β1a and β1b) was required, likely because β1a alone does not always constitute a stable, well-folded domain^16^,^21^. Thus, to test the interaction between CdnL_Cc_ and RNAPβ, a fragment encompassing only β1a (β_16-214_) or the whole β1 domain (β_16-523_) was used. Increased β-galactosidase activity with the β_16-523_ fragment (Fig. 3b) suggests that CdnL_Cc_ conserves the interaction with RNAPβ. To corroborate this interaction with RNAP in *C. crescentus*, we performed coimmunoprecipitation experiments with cells expressing CdnL_Cc_-FLAG and, as a control, with equivalent cells expressing untagged CdnL_Cc_ (see Methods). Detection of a band corresponding to RNAPβ by immunoblot analysis of cells expressing CdnL_Cc_-FLAG confirmed the CdnL_Cc_-RNAP interaction in *C. crescentus* (Fig. 3c).

### CdnL_Cc_ interacts nonspecifically with dsDNA

CdnL_Mt_ has been reported to bind DNA nonspecifically *in vitro* through a positively charged patch at its C-terminal domain^39^, although *in vivo* it was not found on the genome in the absence of RNAP^18^. However, direct DNA binding has never been observed for CdnL_Mx_, which was proposed to associate with DNA exclusively via its interaction with RNAP^9^,^16^. CdnL_Mx_ is overall acidic, as is CdnL_Mt_ (Supplementary Table S1), whereas CdnL_Cc_ is basic and could possibly bind to DNA and other polyanions. We therefore compared the DNA binding behaviour of CdnL_Cc_, CdnL_Tt_ (also basic) and CdnL_Mx_ to a 350-bp double-stranded DNA probe that includes the promoter region of *rrnA* (P_*rrnA*_), one of the two rRNA operons in *C. crescentus*. The probe incubated with CdnL_Cc_ or CdnL_Tt_ and without nonspecific competitor DNA present precipitated in the loading well (not shown) suggesting nonspecific DNA binding. In the presence of nonspecific competitor DNA, a smeared retarded band could be discerned at high protein concentrations (5–10 μM) with CdnL_Cc_ or CdnL_Tt_, but not with CdnL_Mx_, but most of the labeled DNA probe remained free (Fig. 3d). We mutated to Ala three basic residues in the C-terminal domain of CdnL_Cc_ (Arg92, Arg93, Arg130) that align with those in the basic patch mentioned above (Supplementary Fig. S1). Binding to the DNA probe was weakened on mutating Arg130, and abolished on mutating both Arg92 and Arg93 (Supplementary Fig. S6a). Similar results were observed with a randomly chosen intragenic DNA probe *in vitro* (Supplementary Fig. S6b). Altogether, these data suggest that CdnL_Cc_ can bind nonspecifically to DNA *in vitro*, largely via electrostatic interactions.

### CdnL_Cc_ depletion impairs rRNA transcription in *C. crescentus*

**Figure 4:**
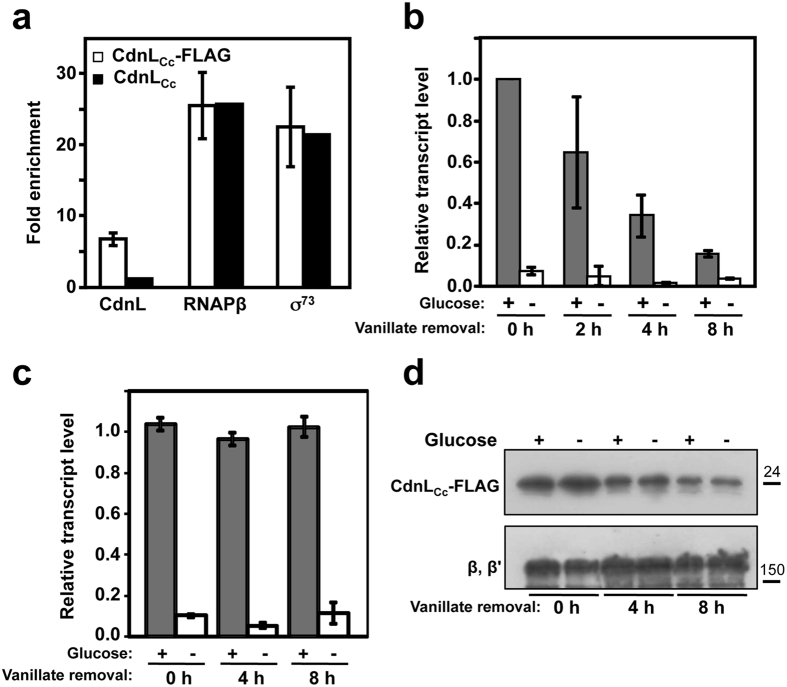
CdnL_Cc_ localizes at an rRNA promoter and affects its transcription *in vivo*. (**a**) CdnL_Cc_ binds to P_*rrnA*_ *in vivo*. ChIP-qPCR analysis using an anti-FLAG antibody on cells expressing CdnL_Cc_-FLAG (strain ME17: Δ*cdnL*_Cc_, P_*van*_::*cdnL*_Cc_-*flag, vanR*; unfilled bars) or CdnL_Cc_ as negative control (strain ME5: Δ*cdnL*_Cc_, P_*van*_::*cdnL*_Cc_, *vanR*; black bars) showing CdnL_Cc_-FLAG enrichment at P_*rrnA*_ *in vivo*, relative to an intergenic region. As positive controls, ChIP-qPCR analysis was carried out using anti-RNAP β or anti-σ^A^ monoclonal antibodies for enrichment of RNAP or σ^73^, respectively, at P_*rrnA*_. (**b**) Effects of CdnL_Cc_ depletion on P_*rrnA*_ promoter activity *in vivo.* CdnL_Cc_ was expressed from the P_*van*_ promoter in strain ME42. Cells grown in M2G with vanillate to OD_660_~0.4 were washed and then grown in vanillate-free medium to block *cdnL*_Cc_ expression, and activity was measured by qRT-PCR at the times indicated. At each time point following vanillate withdrawal, one-half of the sample was washed and resuspended in medium without glucose and the other half remained untreated. Following 15 min incubation at 30 °C, the samples were collected for qRT-PCR analysis. (**c**) qRT-PCR analysis carried out with strain ME40, which expresses CdnL_Cc_-FLAG from the P_*van*_ promoter, using a procedure identical to that in (**b**). Data shown in (**a**–**c**) correspond to the mean and standard error from three biological replicates. (**d**) Immunoblot analysis of CdnL_Cc_-FLAG corresponding to samples in (**c**) with and without glucose deprivation and at the times indicated following vanillate withdrawal (top). As loading control, the same blot was probed using polyclonal anti-RNAP antibodies; the band corresponding to the RNAP β, β′ subunits is shown (bottom). Molecular size markers are shown to the right of the cropped immunoblots by lines and corresponding values in kDa.

Our results thus far indicate that CdnL_Cc_ is required for normal cell growth and that it interacts with RNAPβ, like most of its homologs that have been studied thus far. These have been shown to localize at promoters dependent on the primary σ factor, such as those for rRNA, and to activate them^15^,^16^,^17^,^18^,^19^. Hence, we used quantitative chromatin immunoprecipitation (ChIP) with anti-FLAG antibodies to probe if CdnL_Cc_ localizes to P_*rrnA*_ (the *rrnA* operon promoter region) in *C. crescentus* by using cells expressing CdnL_Cc_-FLAG (strain ME17) or CdnL_Cc_ as negative control (strain ME5). This assay demonstrated that, relative to an intragenic region, CdnL_Cc_ was indeed enriched at P_*rrnA*_, as were the primary σ factor in *C. crescentus* (σ^73^) or RNAPβ used as positive controls (Fig. 4a). We also found that CdnL_Cc_ was enriched at two other σ^73^-dependent promoters but not at one requiring σ^F^, an alternative ECF σ factor in *C. crescentus*^25^,^41^,^42^ (Supplementary Fig. S7). This suggests that CdnL_Cc_, like the homologs studied, is associated with promoters that depend on the primary σ factor.

Next, we examined if CdnL_Cc_ affects transcription *in vivo*, focusing on P_*rrnA*_. We used a *C. crescentus* strain (ME42) that expresses *cdnL*_Cc_ under P_*van*_ control and bears a transcription reporter plasmid with P_*rrnA*_ and the first 81 nucleotides of its leader fused to a *lacZ* gene fragment. This design is based on a previous study^43^, which showed that transcription from this P_*rrnA*_ reporter was rapidly downregulated on glucose starvation, and proposed that this was mediated by an unknown factor. Such a factor could, in principle, be one that activates rRNA transcription but disappears rapidly on glucose starvation to swiftly lower rRNA transcription. CdnL_Cc_ could potentially be this factor if it activates rRNA transcription, and if it becomes unavailable on glucose deprivation. Hence, we tested the effects of depleting CdnL_Cc_ on P_*rrnA*_ transcription *in vivo* and whether this was affected on glucose deprivation.

Cells were grown to mid-log phase with vanillate, after which the inducer was eliminated to restrict *cdnL*_Cc_ expression. This would result in a progressive drop in CdnL_Cc_ levels over time due to intracellular degradation. Reporter P_*rrnA*_ transcription, estimated by qRT-PCR, decreased gradually after removal of vanillate, suggesting that CdnL_Cc_ is directly or indirectly required for P_*rrnA*_ expression (Fig. 4b). Glucose deprivation caused a sharp drop in rRNA transcription, even right after removal of the inducer (Fig. 4b). To test if this is due to rapid degradation of CdnL_Cc_, somehow triggered by glucose starvation, we repeated the above analysis in a strain (ME40) expressing the considerably more stable, yet functional, CdnL_Cc_-FLAG. Rather than gradually decreasing over time, relative transcript levels observed in the presence of glucose now remained fairly steady over the 8 h period (Fig. 4c), consistent with CdnL_Cc_-FLAG persisting even 8 h after removal of the inducer, albeit at lower levels (Fig. 4d). P_*rrnA*_ transcription again dropped dramatically on glucose starvation (Fig. 4c), even though CdnL_Cc_-FLAG levels, at all time points, were comparable with or without glucose deprivation (Fig. 4d). Taken together these results indicate that depletion of CdnL_Cc_ impairs rRNA expression, suggesting that CdnL_Cc_ directly or indirectly promotes rRNA transcription *in vivo*, but that factors and mechanisms other than a rapid loss of CdnL_Cc_ likely determine the sharp fall in rRNA expression occurring on glucose deprivation.

### Missense mutations at conserved CdnL_Cc_ residues cause cold sensitivity

The results thus far establish that CdnL_Cc_ interacts with itself and with RNAPβ, localizes at P_*rrnA*_ and affects its activity. To test if the interaction with RNAPβ is required for CdnL_Cc_ function in *C. crescentus*, two classes of mutations were generated, mimicking those reported previously in other CdnL homologs: mutations in the N-terminal module that disrupt the interaction with RNAP and those in the C-terminal domain that leave the interaction with RNAP intact^16^,^18^,^21^,^22^.

**Figure 5:**
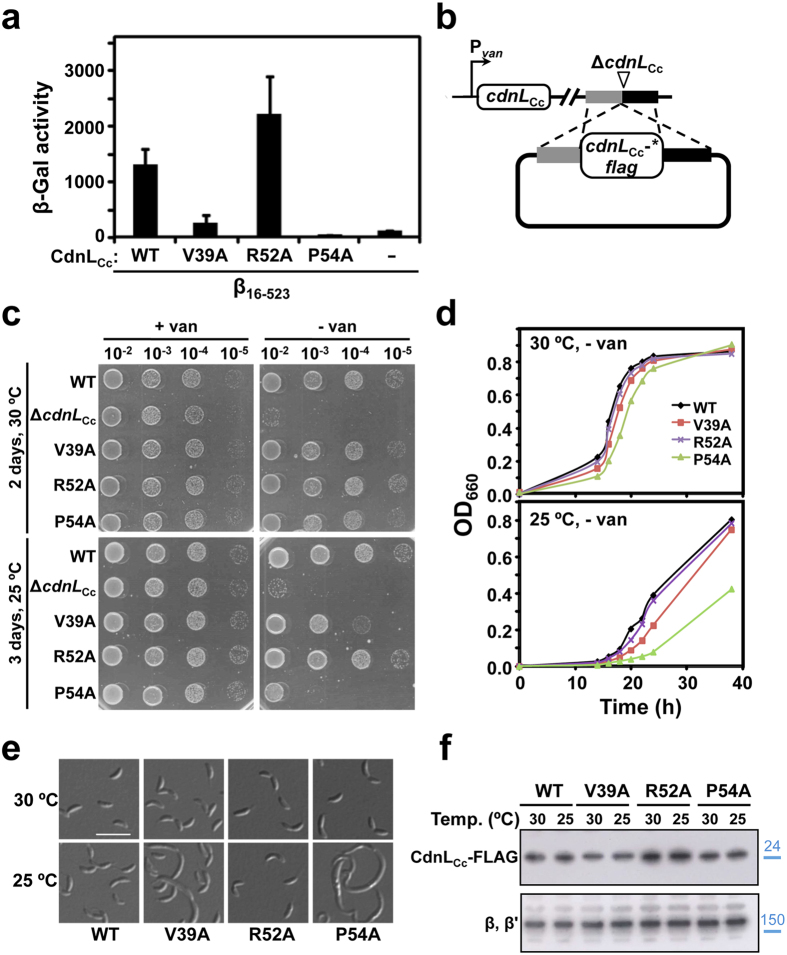
Mutational analysis of CdnL_Cc_-RNAP interaction *in vivo*. (**a**) BACTH analysis of the interaction of CdnL_Cc_ mutants V39A, R52A and P54A (in pKT25) with *C. crescentus* RNAPβ fragment β_16-523_ (in pUT18C). The negative control (–) bears empty pKT25 and pUT18C-β_16-523_. (**b**) Schematic for the strategy employed to check for *cdnL*_Cc_ complementation in *C. crescentus*. A pMR3552 derivative with the required *cdnL*_Cc_-*flag* allele (* indicates mutant) flanked by DNA segments upstream (grey) and downstream (black) of *cdnL*_Cc_ in the genome was introduced into strain ME5, which bears the Δ*cdnL*_Cc_ allele at the endogenous site and P_*van*_-*cdnL*_Cc_ at a heterologous site. Merodiploids resulting from plasmid integration by recombination express both CdnL_Cc_*-FLAG and CdnL_Cc_ in the presence of vanillate (+van) and only the former in the absence of vanillate (−van). (**c**) Complementation analysis in *C. crescentus* of cells bearing the Δ*cdnL*_Cc_ allele or ones expressing at the endogenous site C-terminal FLAG-tagged wild-type CdnL_Cc_ (WT) or the indicated N-terminal CdnL_Cc_ variants. PYE plates with (+van) or without (−van) vanillate were spotted with 8 μl of liquid cultures (OD_660_ ∼ 0.5) at the dilutions indicated and incubated at 30 °C for two days or at 25 °C for three days. (**d**) Growth curves at 30 °C or 25 °C of *C. crescentus* expressing C-terminal FLAG-tagged CdnL_Cc_ (WT) or its indicated variants cultivated in liquid PYE without vanillate using the procedures described in Fig. 1b. (**e**) Cellular morphology examined by DIC microscopy of the wild-type (WT; scale bar: 5 μm) and the indicated mutant cells from (**d**) grown at 30 °C or 25 °C. (**f**) Immunoblot analysis to probe the stability of N-terminal CdnL_Cc_ variants. Cell extracts of strains expressing C-terminal FLAG-tagged CdnL_Cc_ (WT) or its indicated variants grown at 30 °C or 25 °C in PYE with vanillate were probed using anti-FLAG antibodies (top). As loading control, the same blot was probed using polyclonal anti-RNAP antibodies; the band corresponding to the RNAP β, β′ subunits is shown (bottom). Molecular size markers are shown to the right of the cropped immunoblots by lines and corresponding values in kDa (in blue).

CdnL_Cc_ N-terminal residues Val39, Arg52, and Pro54 (Supplementary Fig. S1) were mutated to Ala, since equivalent mutations in CdnL_Mx_, CdnL_Mt_ or CdnL_Tt_ caused loss of interaction with RNAPβ and, where tested, impaired cell growth^16^,^22^,^39^. BACTH indicated significantly reduced interaction of β_16-523_ with mutants V39A and P54A, but not with R52A (Fig. 5a), indicating that CdnL_Cc_ conserves at least two of the expected contacts with RNAPβ. To test the effect of the mutations in *C. crescentus*, plasmids bearing a given allele (expressing the protein with a C-terminal FLAG tag) flanked by ∼500 bp genomic DNA upstream and downstream of *cdnL*_Cc_ were introduced into strain ME5 (Fig. 5b). Transformants with plasmids integrated by homologous recombination at the endogenous *cdnL*_Cc_ site were isolated in the presence of vanillate and then examined after removal of the inducer. On plates lacking vanillate, all three mutants grew normally at 30 °C (Fig. 5c). Notably, V39A and P54A functioned in *C. crescentus* despite their inability to interact with RNAPβ. By contrast, such mutations were lethal in *M. xanthus*^16^ and in *M. tuberculosis*, but apparently not in *M. smegmatis*, closely related to *M. tuberculosis*^21^. Interestingly, we noticed that lowering the growth temperature from 30 °C to 25 °C under vanillate-free conditions caused significant growth arrest of V39A and P54A, while R52A, which continues to interact with RNAPβ, still grew normally (Fig. 5c). Consistent results were obtained for growth in liquid media without vanillate (Fig. 5d), with several cells of the poorly growing V39A and P54A mutants at 25 °C having the aberrant elongated cell morphology of a Δ*cdnL*_Cc_ strain (Fig. 5e). All of the mutant proteins were stable *in vivo* at both growth temperatures (Fig. 5f), implying that loss of function of V39A and P54A at the lower temperature does not stem from protein instability. This behavior does not appear to be caused by increased stability of CdnL_Cc_ due to the C-terminal FLAG tag, since the cold sensitivity was also observed with the representative untagged P54A mutant (Supplementary Fig. S8). Thus, interaction with RNAPβ does not appear to be critical for CdnL_Cc_ function in *C. crescentus* except at lower than standard growth temperatures.

We next examined the effect of mutating Trp90, Arg92, Arg93, Tyr127 or Arg130 (Supplementary Fig. S1) in the CdnL_Cc_ C-terminal domain, which is markedly basic and shares low sequence identity with its acidic counterparts in CdnL_Mx_, CdnL_Mt_ and/or CdnL_Tt_ (Supplementary Table S1). Nonetheless, CdnL_Cc_ residues Trp90, Arg93, and Arg130 are conserved in all four homologs, Arg92 is conserved in CdnL_Mx_ and CdnL_Mt_, and Tyr127 is a Phe in CdnL_Mx_. With CdnL_Mx_, CdnL_Mt_ or CdnL_Tt_, mutational data had shown these residues to be functionally important in at least one of the homologs; and high-resolution structures revealed the residues to be part of a solvent-exposed basic-hydrophobic patch^16^,^18^,^39^,^40^. Moreover, in the crystal structure of the RP_o_-CdnL_Tt_ complex, Trp86 was in a position to interact with a highly conserved thymine (T_12_) at the upstream edge of the DNA bubble, and act as a wedge to prevent bubble collapse; and CdnL_Tt_ activity *in vitro* was shown to require Trp86 and T_12_^19^. Interestingly, mutating this highly conserved tryptophan impaired growth in *M. tuberculosis* but not in *M. smegmatis*^44^ or in *M. xanthus*^16^.

**Figure 6:**
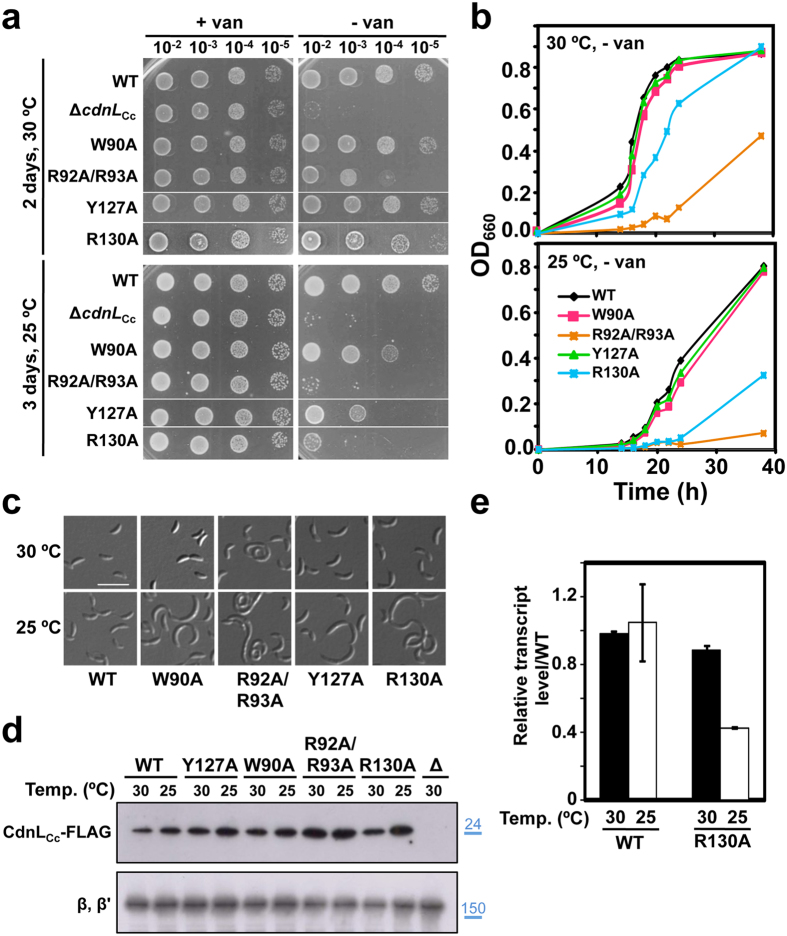
Analysis of C-terminal CdnL_Cc_ mutations *in vivo*. (**a**) Complementation analysis in *C. crescentus* of cells bearing the Δ*cdnL*_Cc_ allele or ones expressing at the endogenous site C-terminal FLAG-tagged wild-type CdnL_Cc_ (WT) or the indicated C-terminal CdnL_Cc_ variants. The analysis was carried out using the same procedures and conditions described in Fig. 5c. (**b**) Growth curves at 30 °C or 25 °C of *C. crescentus* strains expressing the C-terminal FLAG-tagged CdnL_Cc_ (WT) or its indicated variants cultivated in liquid PYE without vanillate using the procedures described in Fig. 1b. (**c**) Cellular morphology examined by DIC microscopy of the wild-type (WT; scale bar: 5 μm) and the indicated mutant cells from (**b**) grown at 30 °C or 25 °C. (**d**) Immunoblot analysis to probe the stability of C-terminal CdnL_Cc_ variants. Cell extracts of strains expressing C-terminal FLAG-tagged wild-type CdnL_Cc_ (WT) or the indicated CdnL_Cc_ mutants grown at 30 °C or 25 °C in PYE with vanillate were probed using anti-FLAG M2 antibodies (top). The negative control “Δ” corresponds to the strain (ME5) with the Δ*cdnL*_Cc_ allele at the endogenous site and expressing untagged CdnL_Cc_ under P_*van*_. As loading control, the same blot was probed using polyclonal anti-RNAP antibodies; the band corresponding to the RNAP β, β′ subunits is shown (bottom). Molecular size markers are shown to the right of the cropped immunoblots by lines and corresponding values in kDa (in blue). (**e**) P_*rrnA*_ promoter activity *in vivo* at 30 °C or at 25 °C in cells expressing CdnL_Cc_-FLAG (WT) or its variant with the R130A mutation under P_*van*_ control (strains ME40 and ME38, respectively). Cells grown overnight at 30 °C in M2G with vanillate were diluted into the same medium to OD_660_ ∼ 0.1. One-half was grown at 30 °C and the other at 25 °C to OD_660_ of 0.3–0.4, and RNA was quantitated using qRT-PCR. Data shown correspond to the mean and standard error from three biological replicates.

The CdnL_Cc_ C-terminal mutants tested grew normally at 30 °C in the absence of vanillate, except for the double R92A/R93A mutant, which did show a growth defect (Fig. 6a). In comparison, the *M. xanthus* mutant corresponding to CdnL_Cc_ R92A/R93A also grew very poorly while that equivalent to Y127A showed somewhat impaired growth^16^, and single mutations of a number of these residues caused lethality in *M. tuberculosis* but not in the closely related *M. smegmatis*^44^. Lowering the *C. crescentus* incubation temperature to 25 °C exacerbated the phenotype of the R92A/R93A mutant, caused somewhat deficient growth of W90A and Y127A, and a marked decrease in growth for R130A (Fig. 6a). Consistent results were obtained in liquid cultures lacking vanillate (Fig. 6b), and several cells of the mutants growing poorly at 25 °C had the abnormal elongated morphology (Fig. 6c). Again, the cold sensitivity does not appear to be due to protein instability (Fig. 6d), or entirely to protein stabilization resulting from the presence of the C-terminal tag, since the R130A mutant without the tag remained cold sensitive, albeit to a lower extent (Supplementary Fig. S8). Relative levels of reporter rRNA estimated by qRT-PCR were comparable for R130A and wild type at 30 °C but dropped significantly for the mutant at 25 °C (Fig. 6f), suggesting that impaired growth correlates with diminished rRNA transcription.

In sum, our data suggest that both classes of functionally important mutations are conserved in CdnL_Cc_ but they cause cold-sensitive phenotypes in *C. crescentus*.

## Discussion

Experimental validation of function for different proteins of the same family is required not only to gain insights into structure-function relationships but also because of several examples of apparently sequence-related proteins shown to be functionally distinct. Thus, CarD and CdnL have different functions, despite various shared structural features and interactions. CdnL_Mx_ and CdnL_Mt_ are similar in their essentiality, structure, and function in stabilizing RP_o_ formation and activating rRNA transcription, whereas CdnL_Bs_ lacks these properties. The fact that nearly all alphaproteobacteria have a CdnL homolog, with those of *C. crescentus* and a few other alphaproteobacteria being listed as essential based on genome-wide Tn-Seq studies^29^,^30^,^31^, prompted us to study CdnL_Cc_. We have shown here that CdnL_Cc_ is not essential, unlike CdnL_Mt_ and CdnL_Mx_, but that its depletion leads to slow growth and cell filamentation in *C. crescentus*. The slow growth probably explains why CdnL_Cc_ was listed among the essential *C. crescentus* proteins^29^, just as in the case of SsrA, GcrA and CcrM, which were initially deemed to be essential in *C. crescentus* but subsequently shown to be dispensable^45^,^46^,^47^. Despite this key difference from its myco/myxobacterial homologs, CdnL_Cc_ nevertheless conserves the interaction with RNAP and other functional determinants, and directly or indirectly affects rRNA transcription, reinforcing the evidence for an important, broadly conserved regulatory role of CdnL in bacteria.

Both *cdnL*_Mx_ and *cdnL*_Mt_ are expressed from a primary σ-dependent promoter with a short 5′-UTR. While *cdnL*_Mt_ is sharply and quickly upregulated upon starvation or exposure to some other stresses^10^, this has not been observed for *cdnL*_Mx_^9^. On the other hand, *cdnL*_Bs_ and *B. burgdorferi cdnL* expression was reportedly induced at lower temperatures from promoters that remain uncharacterized^11^,^48^. We found that expression of *cdnL*_Cc_ increases during exponential growth and appears to be maintained in stationary phase. Global 5′-RACE data for *C. crescentus*^25^ and *S. meliloti*^28^ list their *cdnL* homologs among those genes dependent on the primary σ factor, but unlike *cdnL*_Mx_ or *cdnL*_Mt_, both alphaproteobacterial *cdnL* have 5′-UTRs >200-bp long. The *S. meliloti cdnL* 5′-UTR was reported to contain also a heat-shock σ^H^ promoter^28^,^49^, but we did not find any appreciable change in *cdnL*_Cc_ expression upon heat shock (Supplementary Fig. S9), suggesting absence of a σ^H^ promoter in the *cdnL*_Cc_ 5′-UTR. We found that the intergenic region at the 5′ end of *cdnL* tends to be unusually long in alphaproteobacteria: >200 bp for >60% of the homologs with an overall median of 312 bp. Short 5′ intergenic sequences are only found in the Rickettsiales, which are well known for reductive evolution^50^. A long 5′-UTR may therefore be a common feature for *cdnL* in alphaproteobacteria. Future studies could shed light on the exact role of the long 5′-UTR, if any, on *cdnL*_Cc_ expression.

Our data indicate that CdnL_Cc_ is targeted for proteolysis *in vivo* in a manner dependent on its C-terminal AA motif, and is stabilized against intracellular degradation if this motif is masked by a C-terminal tag or is mutated to DD. This is a characteristic of many ClpXP-dependent substrates, and CdnL_Cc_ degradation was reduced about two-fold upon expressing a dominant-negative ClpX variant that is known to inhibit ClpXP activity^35^,^36^. While this suggests that CdnL_Cc_ may be degraded in a ClpXP-dependent manner in *C. crescentus*, we cannot rule out other mechanisms also operating, such as through the ClpAP protease, as has been observed for other proteins^35^. CdnL_Cc_ levels (in cells expressing FLAG-CdnL_Cc_ under P_*van*_ control) were barely detectable in non-replicative SW cells but easily detectable in ST and PD cells, suggesting that CdnL_Cc_ may be subject to cell cycle-dependent proteolytic control, with a possible role in actively dividing cells. Interestingly, changes in CdnL_Cc_ levels during the cell cycle are akin to those reported for the cell division protein FtsZ, a ClpXP and ClpAP substrate that also becomes stabilized if its C-terminus is tagged or mutated to DD^35^. We found that >80% of the alphaproteobacterial CdnL homologs have a C-terminal AA or VA, including those found in early diverging groups like the Rickettsiales. Hence, proteolysis of CdnL might be a common and conserved ancestral mechanism in alphaproteobacteria. CdnL_Mt_, with a C-terminal AAAS has been shown to be a target of the essential Clp protease in *M. tuberculosis*, a phylum distinct from alphaproteobacteria^51^, suggesting that Clp-mediated CdnL turnover might be a general feature. If so, this would hint at a crucial role for degradation of CdnL for post-translational control of its intracellular levels. However, it appears that degradation of CdnL_Cc_ is not required for normal growth under standard conditions, but might instead be more important under some other untested conditions. Increased intracellular levels of CdnL in *M. tuberculosis*^51^ or *M. xanthus*^9^ are not harmful either. Cells thus appear to cope well with high CdnL concentrations under standard growth conditions.

Depleting CdnL_Cc_ correlates with decreasing rRNA transcription and hence rapid degradation of CdnL_Cc_ could, in principle, be a mechanism for the sharp drop in rRNA transcription that occurs *in vivo* upon glucose starvation in *C. crescentus*^43^. Our finding that this decrease occurs even when CdnL_Cc_ is present at significant levels (due to dysregulation of its proteolysis) points to other mechanisms, as yet unidentified, for downregulating rRNA expression upon glucose limitation. An interesting parallel to this has been reported for *E. coli* DksA, which is a ClpXP substrate^52^ that inhibits rRNA transcription. Here, stabilization and a rapid accumulation of DksA on starvation could in theory swiftly reduce rRNA expression, but this is actually achieved by modulating the amounts of small cofactors like (p)ppGpp and NTPs^53^.

An interesting finding of our study is the cold-sensitive phenotype associated with missense mutations in CdnL_Cc_. Cold sensitivity was observed with N-terminal CdnL_Cc_ mutations that disrupt interaction with RNAPβ, as well as with C-terminal ones that retain this interaction. By contrast, equivalent mutations in CdnL_Mx_ or CdnL_Mt_ impaired the essential function of CdnL even under standard growth conditions^16^,^21^,^44^. *In vitro* and structural studies have linked CdnL function to RP_o_ stabilization by preventing collapse of the transcriptional bubble^15^,^16^,^17^,^18^,^19^. Defective RP_o_ stabilization by CdnL mutants with the N-terminal mutations could be attributed to their lack of RNAP-binding and hence poor recruitment to RP_o_ (or RP_c_), and by CdnL mutants with C-terminal mutations to their inability to accelerate DNA opening and inhibit bubble collapse (they interact with RNAP and are recruited to RP_o_ and RP_c_ like wild-type CdnL)^17^. Since CdnL_Cc_, like CdnL_Mt_ or CdnL_Mx_, binds to an rRNA promoter and affects its expression *in vivo*, it may also function in stabilizing RP_o_ formation. If so, temperature-dependent effects on RP_o_ formation and intrinsic differences between CdnL homologs and/or RNAP could possibly underlie the cold sensitivity displayed by CdnL_Cc_ point mutants. Lower temperatures are known to disfavor RP_o_, which forms *via* a series of conformational changes, starting from RP_c_ assembly to melting of the promoter region between positions −11 and +2 relative to the TSS^2^,^3^. Also, the stabilizing effect *in vitro* of CdnL_Mt_ on RP_o_ is more noticeable at 25 °C than at lower (10 °C) or higher (37 °C) temperatures, presumably because at 25 °C the energy landscape between RP_c_ and RP_o_ is more balanced and the additional binding energy provided by CdnL_Mt_ significantly drives the equilibrium towards RP_o_ formation^15^,^17^.

Our analysis highlights differences between CdnL_Cc_ and its homologs, consistent with CdnL-RNAP interactions being species-specific^16^, but how these translate into the observed cold-sensitive phenotype remains elusive. Significant differences in the stability of the complexes formed by different RNAP on the same promoters reveal mechanistic differences across bacterial species. Thus, the occurrence of CdnL has been correlated to the cognate RNAP forming an unstable RP_o_ and, tellingly, CdnL is absent in *E. coli* whose RNAP appears to form more stable RP_o_ than mycobacterial RNAP^15^. Relative to *M. xanthus* and mycobacteria, *C. crescentus* RNAP could form intrinsically more stable RP_o_, such that lower temperature, on its own unfavorable for RP_o_ formation, is necessary to detect the debilitating effects of CdnL_Cc_ mutations on RP_o_ stabilization. *In vitro* analyses using purified *C. crescentus* RNAP and CdnL_Cc_, an aim for future work, should elucidate this further. Species-specific variations will necessarily have to be invoked to rationalize why *B. subtilis*, with an RNAP that forms unstable RP_o_^54^, has a CdnL that is not essential and that does not bind to RNAP or regulate rRNA transcription^12^,^13^.

In conclusion, our study indicates that *C. crescentus* CdnL is not essential but is nonetheless required for normal growth and morphology, and is likely to be involved in rRNA transcription. The functional role of CdnL therefore appears to be fairly conserved in bacteria. It also highlights species-specific mechanistic differences for this factor relative to some of its homologs, within an otherwise preserved mode of action, that will be useful in understanding the structure-function relationships governing this class of important, RNAP-binding transcriptional regulators.

## Methods

### Strains, plasmids, and growth conditions

Strains and plasmids used in this study are listed in Supplementary Tables S2 and S3, respectively. Growth conditions, and strain and plasmid construction are detailed in Supplementary Methods in the Supplementary information file.

### Bacterial two-hybrid (BACTH) analysis

The *E. coli* BACTH system used is based on functional complementation of the T25 and T18 fragments of the *Bordetella pertussis* adenylate cyclase catalytic domain when two test proteins interact^55^. Coding regions selected were PCR-amplified and cloned into the XbaI and BamHI sites of pKT25, pUT18 or pUT18C (Supplementary Table S3). Given pairs of pKT25 and pUT18/pUT18C constructs were electroporated into *E. coli* strain BTH101 (*cya*^−^), a pair with a vector lacking an insert was the negative control. Interaction was assessed from measurements (mean and standard error of at least three experiments) of β-galactosidase specific activity (β-gal activity, in nmol of *o*-nitrophenyl β-D-galactoside hydrolysed/min/mg protein) from liquid cultures, as described^56^.

### Protein purification

His_6_-tagged CdnL_Cc_ and its variants were overexpressed from pET15b (Novagen) constructs as soluble, native proteins using procedures described for CdnL_Mx_ and CdnL_Tt_^16^. After thrombin digestion to remove the His_6_-tag, the sample was passed through a phosphocellulose column equilibrated with 100 mM NaCl, 50 mM phosphate pH 7.5, 2 mM β-mercaptoethanol, eluted at 0.4 M NaCl, purified by size-exclusion (Superdex200, GE Health Sciences), and concentrated with Amicon Ultra (10000 MWCO from Millipore). Protein concentrations were estimated from absorbance at 280 nm using ε_280_(M^−1^cm^−1^) determined from the sequence (http://web.expasy.org/protparam/).

### Microscopy

At different times during growth, samples were withdrawn and the fluorescent dye 4′-6-diamino-2-phenylindole (DAPI; 350 nm excitation maximum, 461 nm emission maximum) was added to a final concentration of 1 ng/μl. A 1 μl drop applied on 1% agarose pads of M2 salts was examined under a Nikon Eclipse 80i microscope equipped with a Plan Apo VC 100×/1.40 oil immersion objective, and a Hamamatsu ORCA-AG CCD camera. A Nikon UV-2E/C filter set was used for DAPI fluorescence. Images were processed with Metamorph 4.5 (Universal Imaging Group) and Photoshop 6.0 (Adobe Systems).

### Western blot and *in vivo* degradation analysis

Immunoblot analysis in whole cell extracts of CdnL_Cc_ tagged with the FLAG epitope was carried out using standard procedures^9^. Total protein was estimated prior to the analysis and aliquots with equal amounts of protein were resolved in 10% SDS-PAGE gels, transferred to Hybond-ECL membranes, and probed using the ECL system and anti-FLAG M2 monoclonal antibodies (F3165, Sigma-Aldrich). As loading control, the same blot was probed for RNAP (subunits β/β′) using polyclonal *B. subtilis* RNAP holoenzyme antibodies^57^. To test the possibility that CdnL_Cc_ is degraded in a ClpX-dependent manner *in vivo*, we employed a previously established protocol^35^. Basically, the *C. crescentus* strain was grown in PYE with appropriate antibiotics and 0.5 mM vanillate overnight at 30 °C, diluted to an OD_660_ = 0.1 into 20 ml of the same medium, grown to an OD_660_ of 0.4 and divided into two 10 ml cultures. To one, glucose was added to 0.2% final concentration and, to the other, 0.3% xylose to induce clpX*. After 2 h, cells were harvested by centrifugation, washed with 10 ml inducer-free PYE, and suspended in 10 ml fresh inducer-free media. Aliquots (1 ml) withdrawn at 20-min intervals were subjected to immunoblot analysis using anti-FLAG M2 antibodies. Relative band intensities were quantified by densitometry using the ImageJ software program and recommended protocols (NIH). Briefly, bands plus background were selected and a profile plot was obtained for each band (peaks). The straight-line tool was used to minimize background noise by closing off each peak above the baseline of the corresponding profile plot and to adjust the closing at the base of the peak in the case of spill-over signals, and the wand tool was used to quantify the closed peaks. The average and standard error of three independent experiments were used in further analysis. The slope of a linear fit to a plot of the natural logarithm of the relative band intensity (in % normalized to the zero time point) versus time using Sigmaplot (Systat Software Inc) yielded the decay rate and error, and the half-life was determined as ln(2)/-rate. To examine changes during the cell cycle (see Supplementary Methods), aliquots were withdrawn from synchronized cell cultures expressing FLAG-CdnL_Cc_ (strain ME24 grown in the presence of 0.5 mM vanillate) every 20 min, inspected by microscopy for progression of the cell cycle, and analyzed in Western blots using anti-FLAG M2 or anti-CtrA antibodies^38^, as a control for the synchronization protocol.

### Electrophoretic mobility shift assays (EMSA)

EMSA was carried out as described previously^16^. EMSA samples (20 μl) contained 1 nM ^32^P-5′-end radiolabeled, double-stranded DNA probe (13,000 cpm) obtained by PCR and 5 or 10 μM protein in EMSA buffer (80 mM KCl, 25 mM Tris pH 8.0, 5 mM MgCl_2_, 1 mM dithiothreitol, 10% glycerol, 200 ng/ml bovine serum albumin) with 1 μg of poly[dG-dC] or poly[dI-dC] as nonspecific competitor, as indicated. Samples were incubated for 30 min at 37 °C and electrophoresed at 200 V for 1.5 h in 4% nondenaturing PAGE gels in TBE buffer (45 mM Tris-boric acid, 1 mM EDTA) at 10 °C, after which the gel was vacuum dried and analyzed by autoradiography.

### RNA isolation and qRT-PCR analysis

Cells expressing P_*van*_*-cdnL*_Cc_ or P_*van*_*-cdnL*_Cc_-*flag* were grown in 10 ml M2G medium with appropriate antibiotics and 0.5 mM vanillate (M2G-vanillate) overnight at 30 °C. 1 ml of this was innoculated into fresh 50 ml M2G-vanillate and grown to an OD_660_ = 0.3–0.4. Two 4 ml aliquots were withdrawn (time “0”) and pelleted. One was stored at −80 °C until further processing. The other was washed twice with M2-vanillate (lacking glucose) to eliminate the glucose, resuspended in 4 ml M2-vanillate and grown at 30 °C for 15 min, then pelleted and stored at −80 °C until use (time “0”, no glucose). The rest of the 50 ml culture was washed twice with M2G to eliminate vanillate, resuspended in 42 ml of fresh M2G with appropriate antibiotics and incubated at 30 °C. 4 ml aliquots were withdrawn at specific times (2, 4, 8 hours) and treated as with time “0” samples with and without glucose starvation. RNA was extracted from each sample using PureLink RNA Mini Kit (Thermo Fisher Scientific), treated with Turbo DNase (Ambion) for 4 h at 37 °C, and purified using the PureLink RNA Mini Kit. RNA levels were assessed by gel electrophoresis and with NanoDrop ND-1000 (Thermo Fisher Scientific) using a 260 nm extinction coefficient of 40 ng-cm/μl. 2 μg of total RNA was reverse transcribed into cDNA using random hexamer primers (Promega) and Transcriptor Reverse Transcriptase (Roche) in 20 μl of reaction mix as per manufacturer’s instructions. 1 μl of cDNA was added to 10 μl SYBR Green PCR Master Mix (Applied Biosystems or BioRad), with the required primers (100 nM). Each reaction was performed in triplicate, with a control reaction using equivalent starting volume of RNA to verify absence of contaminating DNA. Primers to amplify an ∼50–150 bp region within each transcript were designed using Primer Express 3.0 software, and qRT-PCR was carried out in a StepOne instrument and software using the 1-Step RT-PCR program cycle without reverse transcription (Applied Biosystems). Melting and dissociation curves were determined from 60–95 °C, 30s and 95 °C, 15s. Primers to quantify 16S rRNA transcription from the P_*rrnA*_::*lacZ* reporter plasmid pMR3769 and of *ruvA* (as endogenous control) are those described previously^43^, and their ratio yields the ‘Relative transcript level’ for each sample. For each primer pair a standard RT-PCR curve was generated for five serial ten-fold dilutions of cDNA, and ones with near 100% efficiency were used.

### Coimmunoprecipitation (CoIP)

Strains ME5 (Δ*cdnL*_Cc_, P_*van*_::*cdnL*_Cc_, *vanR*) and ME17 (Δ*cdnL*_Cc_, P_*van*_::*cdnL*_Cc_-*flag, vanR*) were grown in 500 ml PYE with appropriate antibiotics and 0.5 mM vanillate to OD_660_ of ~0.3, harvested by centrifugation, washed thrice with CoIP buffer (20 mM HEPES pH 7.5, 50 mM NaCl, 20% glycerol), and the pellets frozen at −80 °C until further use. Frozen pellets were resuspended in 5 ml CoIP buffer, incubated with 10 mM MgCl_2_, 50 mg lysozyme and 50 units DNase I (Promega) at 4 °C with shaking for 30 min, lysed with a French press at 16,000 psi, and clarified by centrifugation (18,000 *g*, 4 °C, 5 min). Cleared lysates were incubated with 20 μl of pre-equilibrated anti-FLAG agarose affinity gel (FLAGIPT-1, Sigma-Aldrich) overnight at 4 °C with rotation, washed thrice with CoIP buffer (100 mM NaCl), thrice with wash buffer (50 mM Tris-HCl pH 7.4, 150 mM NaCl) in SigmaPrep spin columns, incubated in 150 μl wash buffer containing 100 μg/ml 3xFLAG peptide for 1 h at 4 °C, and eluted. Samples were analyzed by Western blotting using anti-FLAG M2 (F3165, Sigma-Aldrich) or anti-RNAP β 8RB13 (Thermo Fisher Scientific) monoclonal antibodies. Co-IP results were checked for reproducibility in three independent experiments.

### Quantitative chromatin immunoprecipitation (qChIP)

Cells grown to exponential phase (OD_660_ = 0.4–0.6) in 50 ml M2G-vanillate were cross-linked with 1% final concentration (v/v) formaldehyde for 30 min at room temperature with shaking (100 rpm), quenched with 2.5 ml of 2.1 M glycine, pelleted, washed three times with phosphate-buffered saline, and stored at −80 °C until further use. Cell pellets were resuspended in 200 μl ChIP lysis buffer A (20% sucrose, 50 mM NaCl, 10 mM EDTA, 10 mM Tris pH 8, 1 mg/ml lysozyme), incubated for 30 min at 37 °C and cooled in ice. After mixing with 800 μl ChIP lysis buffer B (150 mM NaCl, 1 mM EDTA, 50 mM HEPES-KOH pH 7.5, 1% Triton X-100, 0.1% deoxycholate, 0.1% SDS, Roche Complete protease inhibitor cocktail), they were sonicated with 12 30s on-30s off cycles in a Bioruptor (Diagenode) to obtain ∼0.5 kb long fragments and clarified by centrifugation. 20 μl of the supernatant was kept aside for the input sample. The rest was added to 30 μl of anti-FLAG M2 (F3165, Sigma-Aldrich), anti-σ^A^ 2G10 (Thermo Fisher Scientific), or anti-RNAP β 8RB13 (Thermo Fisher Scientific) monoclonal antibodies previously immobilized (≥4 h incubation at 4 °C and two washes with PBS containing 5 mg/ml BSA) on protein A magnetic Dynabeads (Life Technologies), and incubated overnight at 4 °C with rotation. The beads were washed twice with ChIP lysis buffer B with 0.15 M NaCl, twice with the same buffer but with 0.5 M NaCl, and twice with wash buffer (250 mM LiCl, 10 mM Tris-HCl pH 8.0, 1 mM EDTA, 0.5% NP-40, 0.5% sodium deoxycholate). After a final wash with Tris-EDTA (TE) buffer, the beads were resuspended in 60 μl TE, 1% SDS, and incubated for 10 min at 65 °C. From this, 40 μl was mixed with 40 μl TE/1% SDS and 2.4 μl proteinase K (20 μg/μl), incubated at 42 °C for 2 h, then at 65 °C for 6 h, and the DNA isolated using the Roche High Pure PCR product Purification kit. The input sample was also subjected to the same cross-link reversal/DNA extraction protocols. qPCR was carried out with SYBR Green reaction mix (BioRad) in 0.1 ml MicroAMP FAST optical 48-well reaction plates and a StepOne qPCR apparatus (Applied Biosystems). Primers used were: 5′-TCCACGGGCGTCTGTTAAG-3′ and 5′-CCCCTCGCGACAATATAACG-3′ for P_*rrnA*_; 5′-TGCTCGTGGACGTCAACAAC-3′ and 5′-GGGCGCATAGCCGAGAT-3′ for an intragenic nonpromoter control region (nucleotides 3542858 to 3542913 of gene CCNA_003364, whose σ^F^-dependent expression is activated by heavy metal stress^42^). Standard curves were obtained for each DNA region of interest with serially diluted input DNA sample and its primer pair. Signal enrichment at each promoter is the ratio of promoter-specific to intragenic signal of the ChIP fractions relative to that for the input sample, and is reported as the mean and standard error from three independent experiments.

### Genome analysis

A database comprising 239 representative complete proteomes of alphaproteobacteria was searched for CdnL homologs using BLASTP and CdnL_Cc_ as a query. Results with an e-value < 0.001 were used for alignments. Protein alignments were performed using MUSCLE v3.8.31^58^, and curated with Gblocks 0.91b^59^ using default parameters. Phylogenetic trees were generated from the Gblocks output with FastTree version 2.1.7^60^ using the Whelan and Goldman amino acid replacement matrix and a Gamma20-based likelihood calculation. The phylogenetic trees were visualized using FigTree v.1.4.2 (http://tree.bio.ed.ac.uk/software/figtree/). 5′ intergenic regions were retrieved from the cognate genomic databases and analyzed independently.

## Additional Information

**How to cite this article**: Gallego-García, A. *et al. Caulobacter crescentus* CdnL is a non-essential RNA polymerase-binding protein whose depletion impairs normal growth and rRNA transcription. *Sci. Rep.* **7**, 43240; doi: 10.1038/srep43240 (2017).

**Publisher's note:** Springer Nature remains neutral with regard to jurisdictional claims in published maps and institutional affiliations.

## Supplementary Material

Supplementary Information
