# Supplementary material for: Caulobacter crescentus CdnL is a non-essential RNA polymerase-binding protein whose depletion impairs normal growth and rRNA transcription
Source: Sci Rep. 2017 Feb 24;7:43240. doi: 10.1038/srep43240 (PMC5324124; doi:10.1038/srep43240)
Supplement: Supplementary Information [file srep43240-s1.pdf]

SUPPLEMENTARY INFORMATION

Caulobacter crescentus CdnL is a non-essential RNA polymerase-binding protein whose depletion impairs normal growth and rRNA transcription

Aránzazu Gallego-García[1], Antonio A. Iniesta[1], Diego González[2†], Justine Collier[2], S. Padmanabhan[3], Montserrat Elías-Arnanz[1]*

Supplementary Methods

Strains, plasmids, and growth conditions. Strains and plasmids used in this study are listed in Supplementary Tables S2 and S3, respectively. *C. crescentus* strains were grown at 30 or 25 °C in the rich PYE medium (0.2% Bactopeptone, 0.1% yeast extract, 1 mM $MgSO_4$, 0.5 mM $CaCl_2$), in minimal M2 medium [6.1 mM Na_2HPO_4, 3.9 mM KH_2PO_4, 9.3 mM NH_4Cl, 500 µM $MgSO_4$, 500 µM $CaCl_2$, 1x $FeSO_4$/chelate (Sigma #F10518) with 0.2% glucose (M2G), or in PYE-1.5% Bacto agar solid medium[61], supplemented with the required antibiotic (in liquid/solid media: kanamycin, Km, at 5/25 µg/ml; tetracycline, Tc, at 1/2 µg/ml; chloramphenicol, Cm, at 1 µg/ml). *E. coli* strains DH5α (for plasmid constructions) and BL21-(DE3) (for protein overexpression) were grown in Luria broth at 37 or 25 °C. For inducing gene expression in *C. crescentus* under the vanillate-dependent system, 0.5 mM vanillate (Fluka) was used[27].

Synchronized *C. crescentus* cultures for cell cycle analysis were obtained by centrifugation in Ludox or Percoll density gradients for large- and small-scale synchronization, respectively, and isolating SW cells using established protocols[62,63]. For large-scale synchronization, cells were grown in 1 l of M2G with antibiotics and vanillate to OD_{660} ~0.3, pelleted (9000 rpm, 15 min, 4 °C), and resuspended in 180 ml of ice-cold M2 salts and 60 ml of Ludox (Sigma-Aldrich). The cell suspension was centrifuged at 9000 rpm for 1 h at 4 °C. The bottom SW band was isolated, washed three times in 20 ml of ice-cold M2 salts and resuspended in warm M2G media with vanillate for growth at 30 °C. Cells were examined by microscopy for cell cycle synchrony and progression every 20 min, and 1 ml aliquots were withdrawn, pelleted and frozen at -20 °C. The ~4 ml culture remaining at the end of the cell cycle assay was used in small-scale synchronization by pelleting at 6000 rpm/4 °C, resuspending in 1 ml of ice-cold M2 solution, pelleting again and resuspending in 900 µl M2, and finally mixing with 900 µl of Percoll (Sigma-Aldrich). The cell suspension was centrifuged

for 20 min at 4 °C and 11000 rpm. The bottom SW band and the upper band (ST cells and a small proportion of PD cells) were isolated, washed three times in 1 ml of ice-cold M2 and frozen at -20 °C. Frozen pellets were thawed and analyzed by Western blot.

Strains with gene deletions, mutations, or epitope fusions were constructed using the two-step allele exchange homologous recombination and a Km^R selection/*sacB* (sucrose sensitivity, Sac^S) protocol. Genomic DNA isolated using the Wizard kit (Promega) was used to PCR-amplify *cdnL*$_{Cc}$. To delete *cdnL*$_{Cc}$ ~500 bp genomic DNA upstream and downstream of the gene was PCR-amplified and cloned into pNPTS138 to generate pMR3552 with, a BamHI site replacing *cdnL*$_{Cc}$ (Supplementary Table S3). The construct, with a Km^R marker for positive selection and Sac^S for negative selection, was introduced into *C. crescentus* by electroporation[61]. Transformants with the plasmid integrated into the chromosome by homologous recombination (merodiploids) were selected on PYE-Km plates, then grown for several generations in the absence of antibiotic and plated on PYE-agar with 3% sucrose to select for haploid cells (against Sac^S merodiploid cells). To obtain strain ME50, with the Δ*cdnL*$_{Cc}$ allele in the absence of a complementing *cdnL* copy, plating was done with increasing dilutions to detect small, slow growing colonies that typically appeared after four-five days of growth, in contrast to the normally growing colonies observed after two days. The latter had the wild-type allele and the former had the Δ*cdnL*$_{Cc}$ allele, as assessed by PCR of genomic DNA isolated from the haploid colonies. To obtain strain JC784, where *cdnL* is replaced with a spectinomycin/streptomycin resistance ($Spec^R$/$Strep^R$) omega (Ω) cassette (Δ*cdnL*$_{Cc}$::Ω), two 500-bp fragments upstream and downstream of *cdnL*$_{Cc}$ were PCR-amplified, digested to yield NheI-BamHI and BamHI-HindIII fragments, respectively, and cloned into pNPTS138 to generate pNPTS138-Δ*cdnL*$_{Cc}$. The Ω cassette was excised by BamHI digestion of pNPT228-*gcrA*P-*lacZ*::Ω and cloned into BamHI-digested pNPTS138-Δ*cdnL*$_{Cc}$ to generate pNPTS138-Δ*cdnL*$_{Cc}$::Ω. The latter was electroporated into the wild-type strain containing a low-copy number plasmid with *cdnL*$_{Cc}$ under the control of

a xylose-inducible promoter (pRXMCS-6-*cdnL*$_{Cc}$) and transformants with the Δ*cdnL*$_{Cc}$::Ω allele identified by PCR, as before. The deletion was then transduced into the wild-type strain (200 μl) using 50 μl phage ϕCR30 stock prepared on the aforementioned strain bearing the Δ*cdnL*$_{Cc}$::Ω allele following standard protocols[61]. Transductants were selected on PYE/Spec/Strep plates (30-50 colonies were obtained) and checked for the absence of Cm^R and *cdnL*$_{Cc}$. For conditional expression of *cdnL*$_{Cc}$ from the vanillate-inducible promoter (P_{van}), the gene was cloned into the NdeI and NheI sites of the integrative shuttle vector pVGFPC-5[27] to generate pMR3572 (Supplementary Table S3).

To generate C-terminally FLAG-tagged fusions, the epitope sequence was fused to the 3′-end of *cdnL*$_{Cc}$ by PCR. Site-directed *cdnL*$_{Cc}$ mutants were obtained by custom gene synthesis (GenScript). Each of these was cloned into the BamHI site of pMR3552 and the resulting construct was electroporated into strain ME5 (Supplementary Table S2) and plated in the presence of 0.5 mM vanillate to express the wild-type *cdnL*$_{Cc}$ copy under P_{van} control. For complementation analysis, cells were grown in PYE with the required antibiotics and vanillate to OD_{660}=0.5, pelleted, washed twice with vanillate-free PYE and then spotted on plates supplied with the required antibiotics and with or without vanillate at the dilutions indicated and incubated at 25 or 30 °C. Promoter analysis of *cdnL*$_{Cc}$ was carried out with a *lacZ* fusion to a 501-bp fragment immediately upstream (positions 748213-748713 in *C. crescentus* NA1000 genome) of *cdnl*$_{Cc}$, which was PCR amplified with 5′-NheI and 3′-BamHI sites, cloned into the pCR™-Blunt II-TOPO vector (Invitrogen), excised with EcoRI and BamHI, and cloned into the corresponding sites on the pJGZ290 vector (Supplementary Table S3). The *C. crescentus* strain with the *lacZ* probe was grown in PYE to various OD_{660}, and specific β-galactosidase activity (β-Gal activity, in nmol of *o*-nitrophenyl β-D-galactoside hydrolysed/min/mg protein) was measured in a SpectraMax 340 microtitre plate reader (Molecular Devices) and reported as the mean and standard error of at least three experiments. The P_{rrnA}::*lacZ* plasmid pMR3769

(Supplementary Table S3), used for qRT-PCR of 16S rRNA transcription, was constructed by fusing the *rrnA* promoter region (spanning positions -570 to +80 relative to the transcription start site) to an *E. coli lacZ* fragment and introduced into the AscI and NheI sites of pXGFPC-2, as described elsewhere[43].

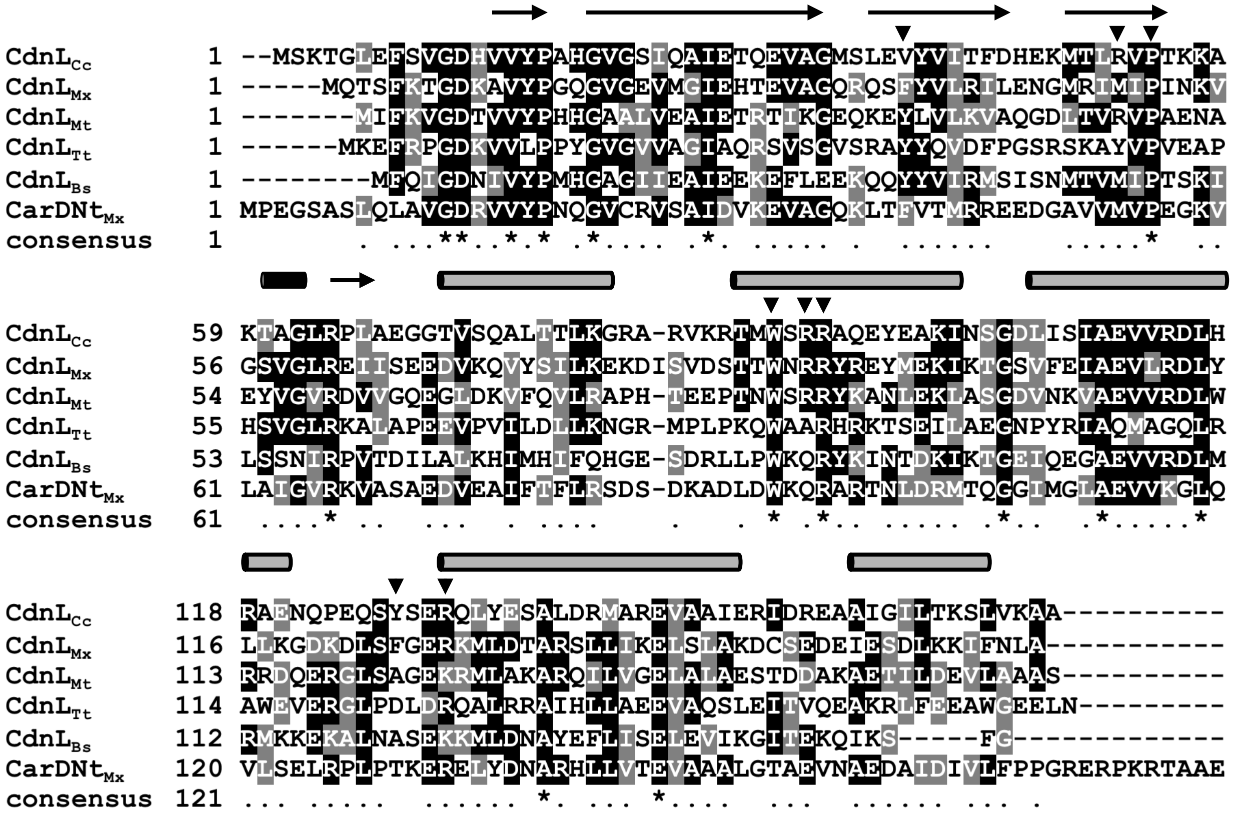

Supplementary Figure S1. Protein sequence comparisons of CdnL homologs. Sequence alignment of $CdnL_{Cc}$, $CdnL_{Mx}$, $CdnL_{Mt}$, $CdnL_{Tt}$, $CdnL_{Bs}$, and $CarDNt_{Mx}$ (the N-terminal domain of *M. xanthus* CarD). Locus tags for *cdnL*$_{Cc}$ are CCNA_00690 in the CB15N/NA1000 genome (GenBank ID CP001340) or CC_0653 in the CB15 genome (GenBank ID AE005673). Residues are shaded black (with an asterisk in the consensus line below) when identical in the majority of the aligned sequences, or grey when similar. Secondary structural elements from the $CdnL_{Mx}$ structure are shown above the sequence with arrows for β-strands, grey rods for α-helices, and the short black rod for a 3_{10}-helix. Arrowheads point to $CdnL_{Cc}$ mutations examined in this study.

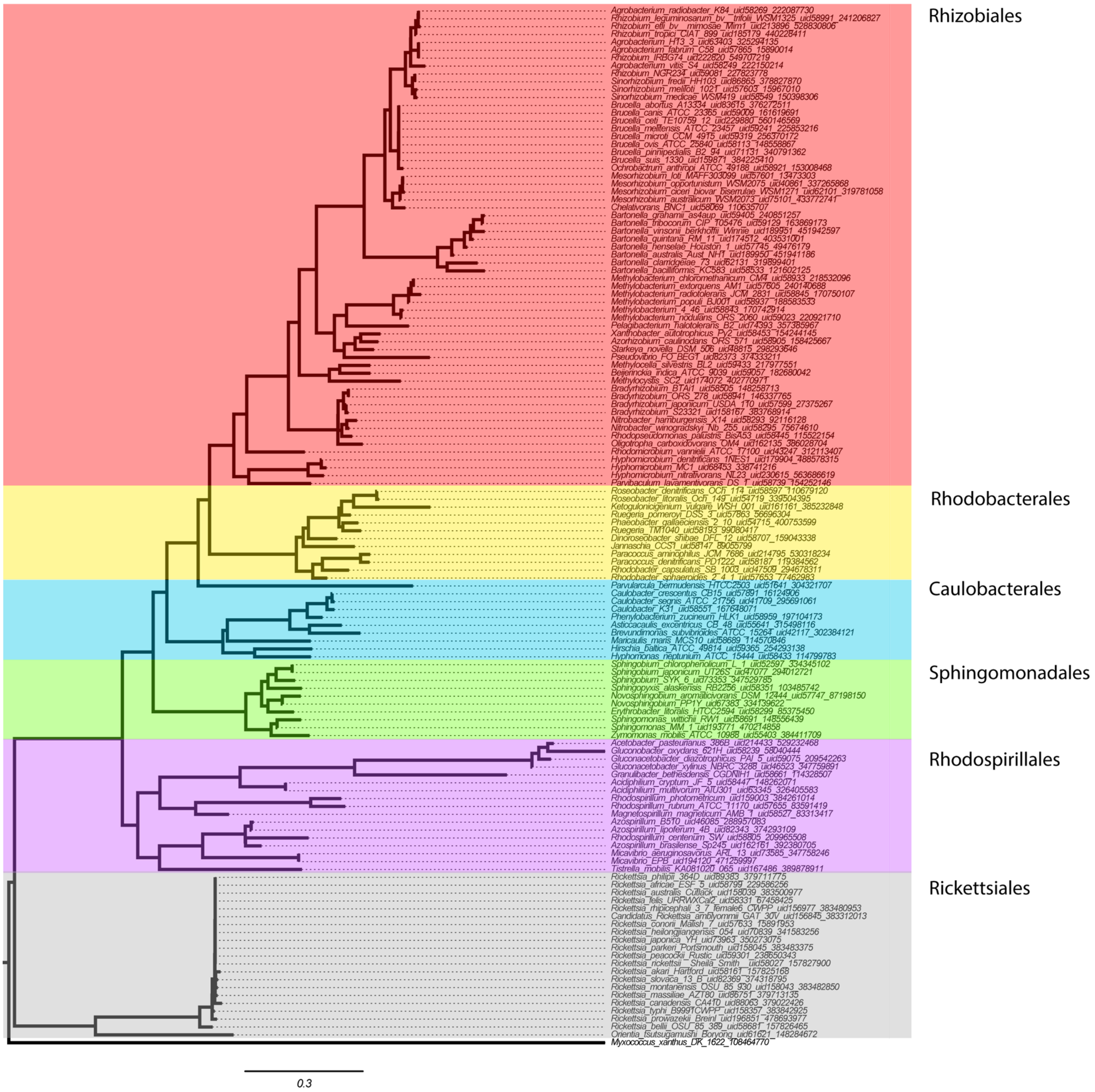

Supplementary Figure S2. **Phylogenetic distribution of CdnL in alphaproteobacteria.** Phylogenetic tree based on a curated multiple alignment of 131 amino acid sequences of alphaproteobacterial CdnL homologs. The *M. xanthus* CdnL homolog was added and used as an outgroup to root the tree. Only one representative of each available alphaproteobacterial species was selected; a small number of chimeric and obviously divergent sequences were excluded. The colors help distinguish the major orders among alphaproteobacteria.

a

TCCCCGCGACGATTTCTCAAGCCCAGAGCGTGACCGCCTCGCGAAAGCTTGGCGGCGTCTCTTTGCG

CGCCAGGACGCAACCCTTTCATAGGGCTTAATTTTATGCTATTGTCTTCGACGACGTGACAGCCGCG

CTCGTTCGCGACGGTCCGTCTTGTCGAAGAAGCCGTTTCAGAAAACCCATTTGAACCGGCCCGCCCG

ATCGAAGGCGAAGGGTGTCTTAGAGGTCTATTCCAAATCCTGACAGGCGTGGCCCCGCGTTCCGTAT

TCCGCGGAGCGGGAACCTGTCGCTTTTGGTTTGGGAATGATGACGGCGTT_CGGACCC_**CGA**_GGGTCCG_

TATTGAAGAGGACGAACATG

b

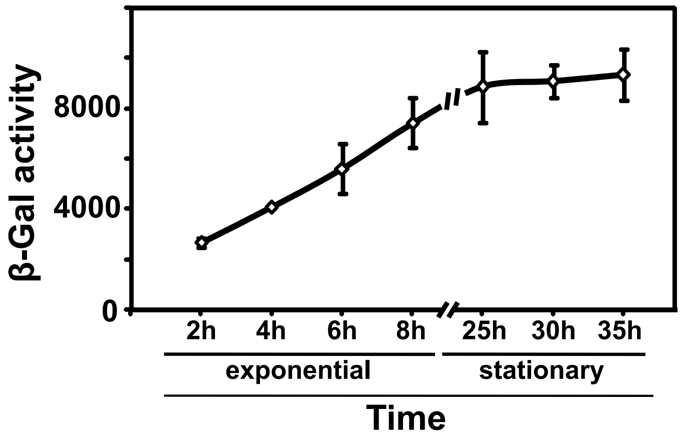

Supplementary Figure S3. **Promoter region and expression levels of *cdnL*$_{Cc}$.** (**a**) DNA sequence of the 352-bp intergenic region upstream of *cdnL*$_{Cc}$. The putative -10 and -35 promoter elements are shaded black, the TSS is in grey and underlined, the initiator ATG codon is boxed and an inverted repeat (that could be a binding site for an unknown transcriptional factor) is in italics and underlined. (**b**). Specific β-galactosidase activity (β-Gal activity) for strain JC735, which bears a *lacZ* reporter fused to a DNA fragment including the intergenic region upstream of *cdnL*$_{Cc}$ shown in (a). β-Gal activity was estimated at various times during exponential growth and stationary phase. The mean and standard error of three independent measurements are shown.

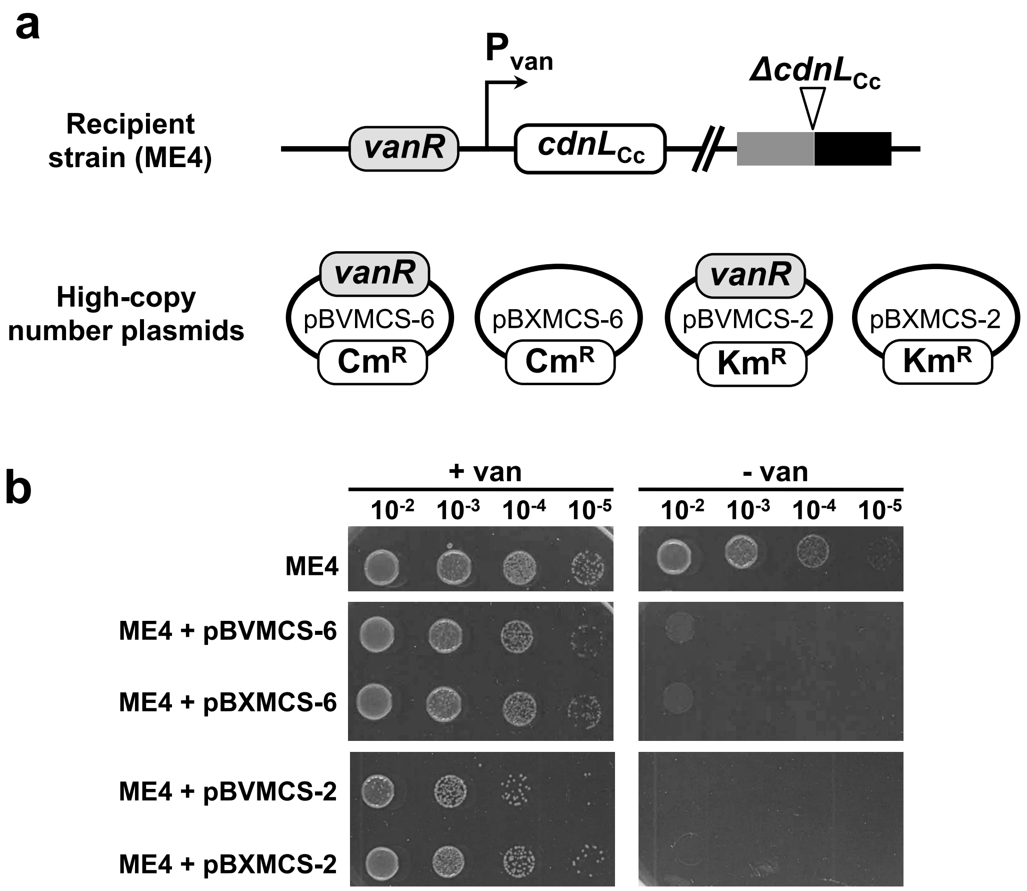

Supplementary Figure S4. Effect of high-copy number plasmids upon CdnL$_{Cc}$ depletion. (**a**) Schematic showing *C. crescentus* strain ME4, bearing an in-frame deletion of the endogenous *cdnL*$_{Cc}$ gene and a vanillate-inducible P$_{van}$-*cdnL*$_{Cc}$ complementing copy, into which the different high-copy number plasmids[27] shown schematically below were introduced. (**b**) Growth of ME4 transformed with different high-copy number plasmids. Liquid cultures (OD_{660}~0.5) were serially diluted, spotted (8 μl) on PYE agar with or without vanillate, and plates were scanned after two days of growth at 30 ºC. ME4 + pBVMCS-6 corresponds to strain ME5 of Fig. 1a.

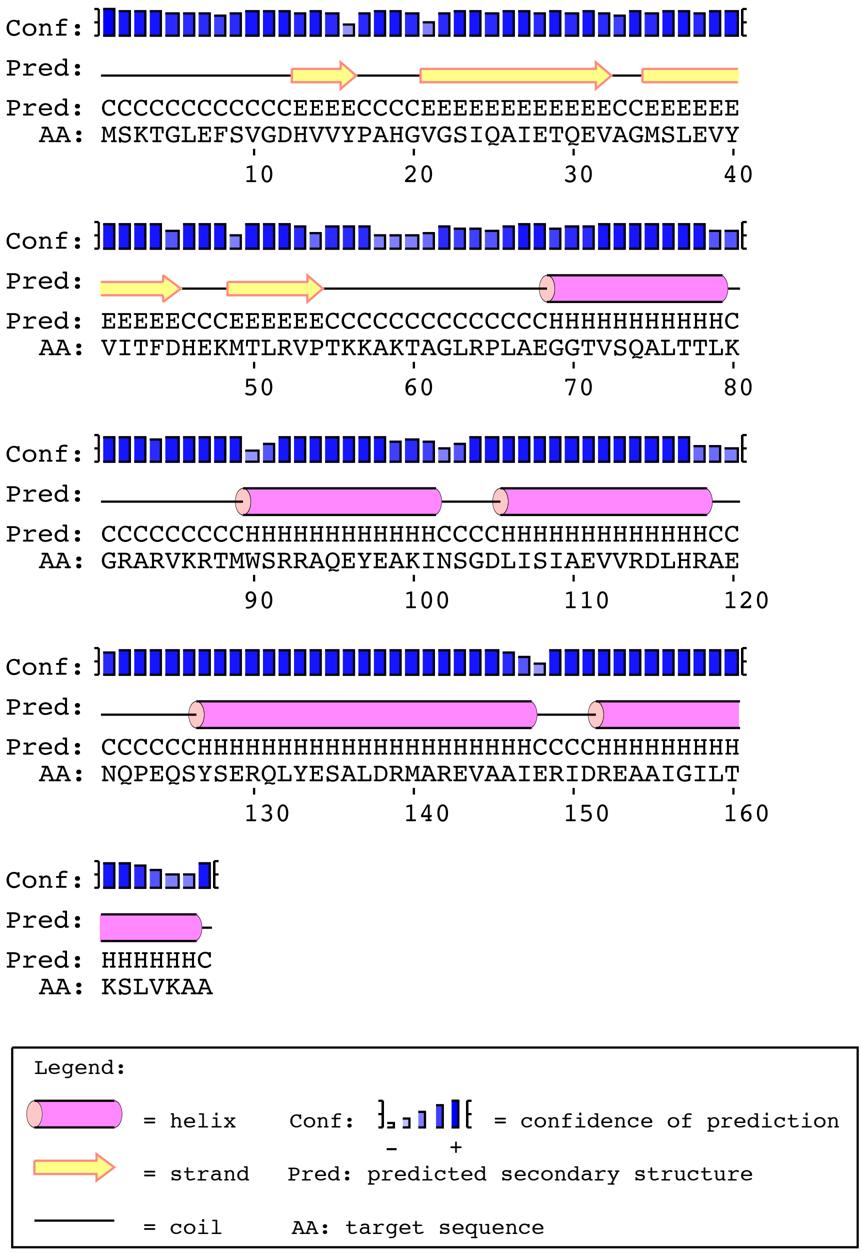

Supplementary Figure S5. **Secondary structure prediction of CdnL$_{Cc}$.** Secondary structure prediction was performed using PSIPRED (http://bioinf.cs.ucl.ac.uk/psipred/). The color code and symbol description are indicated in the box below.

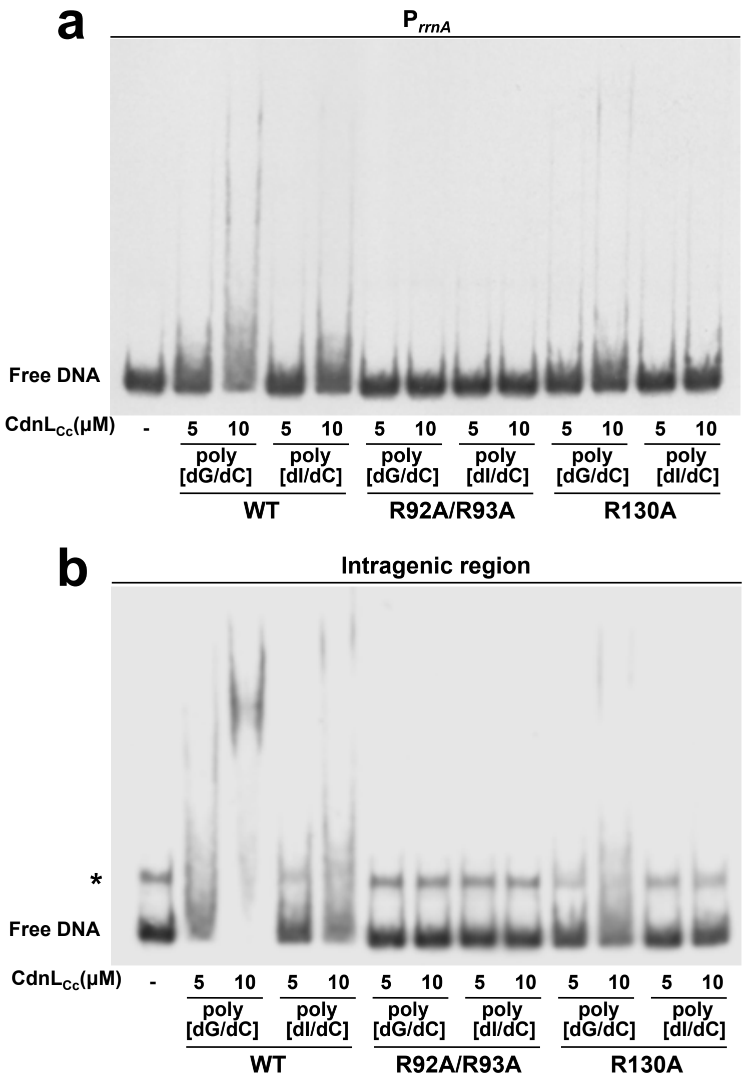

Supplementary Figure S6. **Effect of mutating basic residues in CdnL$_{Cc}$ on its nonspecific DNA binding *in vitro*.** (**a**) EMSA for the DNA binding of CdnL$_{Cc}$ and its R92A/R93A and R130A variants performed with the 350-bp P$_{rrnA}$ DNA probe, as described in Fig. 3d. Protein concentrations and the presence of 1 µg of poly[dG-dC] or poly[dI-dC] as nonspecific competitor were as indicated. (**b**) Same as in (a) but using a 350-bp DNA probe corresponding to a randomly selected intragenic region (within gene CCNA_03365). The asterisk indicates a secondary PCR-amplified product, which was also nonspecifically retarded by CdnL$_{Cc}$.

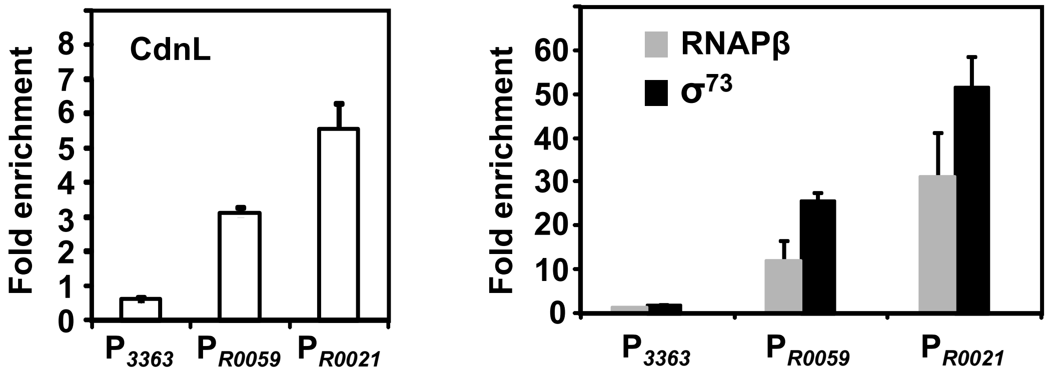

Supplementary Figure S7. CdnL enrichment at various promoters. ChIP-qPCR analysis on cells expressing $CdnL_{Cc}$-FLAG (strain ME17) showing $CdnL_{Cc}$ enrichment *in vivo* (left panel), relative to an intergenic region, at two selected σ^{73}-dependent promoters, P_{R0021} (of the tRNA-Pro gene) and P_{R0059} (of the tRNA-Thr gene), but not at the σ^{F}-dependent promoter P_{3363} (of gene CCNA_3363, encoding a conserved hypothetical membrane protein). The panel on the right shows the corresponding enrichment analysis for σ^{73} and RNAPβ. Primers used in the ChIP-qPCR are: (i) R0059-F and R0059-R for P_{R0059}, and R0021-F and R0021-R for P_{R0021} that have been described by Haakonsen *et al*[41]; (ii) PCCNA3363Fw (5′-TTTTGTTCGCCGATCACTGTAA-3′) and PCCNA3363Rv (5′-CGGTCGTGGGAGAGTTCGT-3′) for the σ^{F}-dependent promoter of gene CCNA_003363.

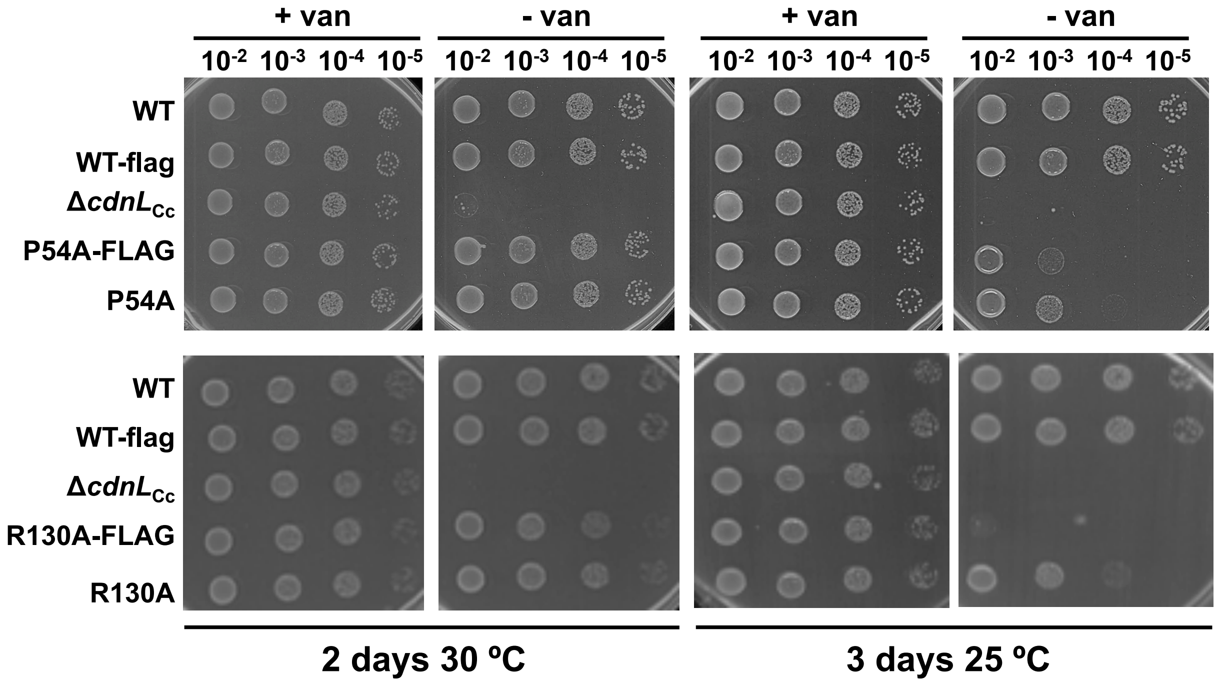

Supplementary Figure S8. Comparison of representative CdnL$_{Cc}$ mutants with and without the C-terminal FLAG tag. Complementation analysis was carried out as in Figs. 5c and 6a.

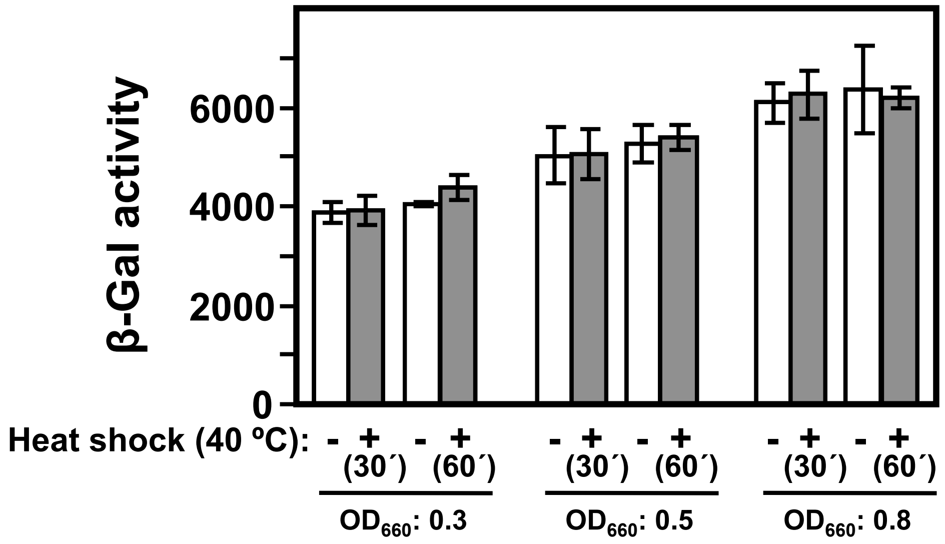

Supplementary Figure S9. ***cdnL*$_{\mathbf{Cc}}$ expression on heat shock treatment.** Specific β-galactosidase activity (β-Gal activity) for strain JC735, which bears the P$_{cdnLCc}$::*lacZ* reporter probe (as in Supplementary Fig. S3), at the indicated OD_{660} during normal growth at 30 °C (unfilled bars) or after a heat shock at 40 °C (grey bars) for 30 min or 60 min. The mean and standard errors of three independent measurements are shown.

Supplementary Table S1. Sequence-based comparative analysis of $CdnL_{Cc}$ and its homologs

**Protein**	**Full-length**		**N-terminal domain**[a]		**C-terminal domain**[a]	
	Size (no. residues) (% Iden./Sim.)[b]	pI[c]	Size (residue no.) (% Iden./Sim.)[b]	pI[c]	Size (residue no.) (% Iden./Sim.)[b]	pI[c]
$CdnL_{Cc}$	167 (100/100)	8.86	1-71 (100/100)	6.25	72-167 (100/100)	9.51
$CdnL_{Mx}$	164 (32/49)	5.39	1-68 (35/43)	5.22	69-164 (30/53)	5.55
$CdnL_{Mt}$	162 (34/51)	5.49	1-66 (38/55)	5.19	67-162 (31/48)	5.88
$CdnL_{Tt}$	164 (26/41)	8.92	1-67 (32/46)	9.52	68-164 (22/38)	6.16
$CdnL_{Bs}$	153 (30/53)	6.92	1-65 (39/62)	4.84	66-153 (31/59)	9.19
$CarDNt_{Mx}$	179 (28/43)	5.21	1-72 (35/43)	5.28	73-179 (24/39)	5.18

[a]Based on $CdnL_{Mx}$ structural analysis[16]. [b]Sequence identity/similarity (in %) relative to $CdnL_{Cc}$. [c]Theoretical pI calculated based on sequence (http://web.expasy.org/protparam).

Supplementary Table S2. ***C. crescentus*** **strains used in this work**

Strain	Description	Source
CB15N /NA1000	Synchronizable derivative of wild-type CB15	62
JC735	P_{cdnLCc}*::lacZ*	pRK290-*cdnL*$_{Cc}$*-UTR* x CB15N
JC784	Δ*cdnL*$_{Cc}$::Ω. $Spec^R$/$Strep^R$	pNPTS138-Δ*cdnL*$_{Cc}$-Ω x CB15N
ME1	*cdnL*$_{Cc}$/Δ*cdnL*$_{Cc}$. Km^R Sac^S	pMR3552 x CB15N
ME2	*cdnL*$_{Cc}$/Δ*cdnL*$_{Cc}$, P_{van}::*cdnL*$_{Cc}$. Tc^R Km^R Sac^S	pMR3572 x ME1
ME4	Δ*cdnL*$_{Cc}$, P_{van}::*cdnL*$_{Cc}$. Tc^R	Haploid from ME2
ME5	Δ*cdnL*$_{Cc}$, P_{van}::*cdnL*$_{Cc}$, *vanR*. Tc^R Cm^R	pBVMCS-6 x ME4
ME8	*cdnL*$_{Cc}$*-flag*/Δ*cdnL*$_{Cc}$, P_{van}::*cdnL*$_{Cc}$, *vanR*. Tc^R Cm^R Km^R Sac^S	pMR3845 x ME5
ME10	*cdnL*$_{Cc}$*(V39A)-flag*/Δ*cdnL*$_{Cc}$, P_{van}::*cdnL*$_{Cc}$, *vanR*. Tc^R Cm^R Km^R Sac^S	pMR3918 x ME5
ME12	*cdnL*$_{Cc}$*(R52A)-flag*/Δ*cdnL*$_{Cc}$, P_{van}::*cdnL*$_{Cc}$, *vanR*. Tc^R Cm^R Km^R Sac^S	pMR3920 x ME5
ME13	*cdnL*$_{Cc}$*(P54A)-flag*/Δ*cdnL*$_{Cc}$, P_{van}::*cdnL*$_{Cc}$, *vanR*. Tc^R Cm^R Km^R Sac^S	pMR3921 x ME5
ME14	*cdnL*$_{Cc}$/Δ*cdnL*$_{Cc}$, P_{van}::*cdnL*$_{Cc}$*-flag*. Tc^R Km^R Sac^S	pMR3879 x ME1
ME16	Δ*cdnL*$_{Cc}$, P_{van}::*cdnL*$_{Cc}$*-flag*. Tc^R	ME14 haploid
ME17	Δ*cdnL*$_{Cc}$, P_{van}::*cdnL*$_{Cc}$*-flag*, *vanR*. Tc^R Cm^R	pBVMCS-6 x ME16
ME20	*cdnL*$_{Cc}$*(Y127A)-flag*/Δ*cdnL*$_{Cc}$, P_{van}::*cdnL*$_{Cc}$, *vanR*. Tc^R Cm^R Km^R Sac^S	pMR4052 x ME5
ME21	*cdnL*$_{Cc}$*(W90A)-flag*/Δ*cdnL*$_{Cc}$, P_{van}::*cdnL*$_{Cc}$, *vanR*. Tc^R Cm^R Km^R Sac^S	pMR4208 x ME5
ME22	*cdnL*$_{Cc}$*(R92A/R93A)-flag*/Δ*cdnL*$_{Cc}$, P_{van}::*cdnL*$_{Cc}$, *vanR*. Tc^R Cm^R Km^R Sac^S	pMR4209 x ME5
ME23	*cdnL*$_{Cc}$*(R130A)-flag*/Δ*cdnL*$_{Cc}$, P_{van}::*cdnL*$_{Cc}$, *vanR*. Tc^R Cm^R Km^R Sac^S	pMR4210 x ME5
ME24	*cdnL*$_{Cc}$, P_{van}::*flag-cdnL*$_{Cc}$, *vanR*. Km^R Cm^R	pMR4389 x pBVMCS-6 x CB15N
ME25	*cdnL*$_{Cc}$, P_{van}:: *cdnL*$_{Cc}$*-flag*, *vanR*. Km^R Cm^R	pMR4390 x pBVMCS-6 x CB15N
ME26	*cdnL*$_{Cc}$, P_{van}::*flag-cdnL*$_{Cc}$*(DD)*, *vanR*. Km^R Cm^R	pMR4396 x pBVMCS-6 x CB15N
ME27	*cdnL*$_{Cc}$, P_{van}::*flag-cdnL*$_{Cc}$, P_{xyl}::*clpX**, *vanR*. Km^R Cm^R Tc^R	pMO88 x ME24
ME28	*cdnL*$_{Cc}$, P_{van}::*cdnL*$_{Cc}$*-flag*, P_{xyl}::*clpX**, *vanR*. Km^R Cm^R Tc^R	pMO88 x ME25
ME29	*cdnL*$_{Cc}$, P_{van}::*flag-cdnL*$_{Cc}$*(DD)*, P_{xyl}::*clpX**, *vanR*. Km^R Cm^R Tc^R	pMO88 x ME26
ME32	*cdnL*$_{Cc}$/Δ*cdnL*$_{Cc}$, P_{van}::*cdnL*$_{Cc}$*(R130A)-flag*. Km^R Sac^S Tc^R	pMR4412 x ME1
ME35	Δ*cdnL*$_{Cc}$, P_{van}::*cdnL*$_{Cc}$*(R130A)-flag*. Tc^R	ME32 haploid
ME38	Δ*cdnL*$_{Cc}$, P_{van}::*cdnL*$_{Cc}$*(R130A)-flag*, P_{rrnA}::*lacZ*, *vanR*. Tc^R Km^R Cm^R	pMR3769 x pBVMCS-6 x ME35
ME39	*cdnL*$_{Cc}$*(DD)*/Δ*cdnL*$_{Cc}$, P_{van}::*cdnL*$_{Cc}$, *vanR*. Tc^R Cm^R Km^R Sac^S	pMR4413 x ME5
ME40	Δ*cdnL*$_{Cc}$, P_{van}::*cdnL*$_{Cc}$*-flag*, P_{rrnA}::*lacZ*, *vanR*. Tc^R Km^R Cm^R	pMR3769 x ME17
ME41	*cdnL*$_{Cc}$/Δ*cdnL*$_{Cc}$, P_{van}::*cdnL*$_{Cc}$, *vanR*. Tc^R Cm^R Km^R Sac^S	pMR4442 x ME5
ME42	Δ*cdnL*$_{Cc}$, P_{van}::*cdnL*$_{Cc}$, P_{rrnA}::*lacZ*, *vanR*. Tc^R Km^R Cm^R	pMR3769 x ME5
ME43	*cdnL*$_{Cc}$*(P54A)*/Δ*cdnL*$_{Cc}$, P_{van}::*cdnL*$_{Cc}$, *vanR*. Tc^R Cm^R Km^R Sac^S	pMR4640 x ME5
ME44	*cdnL*$_{Cc}$*(R130A)*/Δ*cdnL*$_{Cc}$, P_{van}::*cdnL*$_{Cc}$, *vanR*. Tc^R Cm^R Km^R Sac^S	pMR4641 x ME5
ME45	Δ*cdnL*$_{Cc}$, P_{van}::*cdnL*$_{Cc}$. Tc^R Cm^R	pBXMCS-6 x ME4
ME48	Δ*cdnL*$_{Cc}$, P_{van}::*cdnL*$_{Cc}$, *vanR*. Tc^R Km^R	pBVMCS-2 x ME4
ME49	Δ*cdnL*$_{Cc}$, P_{van}::*cdnL*$_{Cc}$. Tc^R Km^R	pBXMCS-2 x ME4
ME50	Δ*cdnL*$_{Cc}$	Haploid from ME1

Km^R, Tc^R, Cm^R: resistance to kanamycin, tetracycline and chloramphenicol, respectively. Sac^S: sensitivity to sucrose. *vanR*: presence of a high-copy number plasmid expressing the VanR repressor.

Supplementary Table S3. Plasmids used in this work

Plasmid	Description	Source
pBVMCS-2	High-copy vector for vanillate-inducible expression. Km^R	27
pBVMCS-6	High-copy vector for vanillate-inducible expression. Cm^R	27
pBXMCS-2	High-copy vector for xylose-inducible expression. Km^R	27
pBXMCS-6	High-copy vector for xylose-inducible expression. Cm^R	27
pET15b	Vector for expression of H_6-tagged proteins	Novagen
pJGZ290	pRK290 with promoter-less *lacZ* gene for promoter-*lacZ* transcriptional fusions	(M.R. Alley & J.W. Gober, unpublished)
pKT25	Vector for C-terminal fusion constructs to the T25 fragment of CyaA for use in bacterial two-hybrid analysis. Km^R	55
pMO88	*clpX* with mutation in ATP binding site (*clpX**) regulated by P_{xyl}	36
pMR3552	pNPTS138 with genomic regions flanking $cdnL_{Cc}$ for complementation analysis. Km^R Sac^S	This work
pMR3572	pVGFPC-5 with $cdnL_{Cc}$ under P_{van} control. Tc^R	This work
pMR3626	pKT25 with $cdnL_{Cc}$. Km^R	This work
pMR3645	pUT18C with the gene for *C. crescentus* RNAP $\beta_{16\text{-}214}$. Amp^R	This work
pMR3701	pUT18C with the gene for *C. crescentus* RNAP $\beta_{16\text{-}523}$. Amp^R	This work
pMR3769	pXGFPC-2 with P_{rrnA}::*lacZ* reporter. Km^R	This work
pMR3795	pUT18 with $cdnL_{Cc}$. Amp^R	This work
pMR3803	pKT25 with $cdnL_{Cc}$*(V39A)*. Km^R	This work
pMR3805	pKT25 with $cdnL_{Cc}$*(R52A)*. Km^R	This work
pMR3806	pKT25 with $cdnL_{Cc}$*(P54A)*. Km^R	This work
pMR3845	pMR3552 derivative with $cdnL_{Cc}$-*flag*. Km^R Sac^S.	This work
pMR3879	P_{van}::$cdnL_{Cc}$-*flag*. Tc^R	This work
pMR3918	pMR3552 with $cdnL_{Cc}$*(V39A)-flag*. Km^R Sac^S	This work
pMR3920	pMR3552 with $cdnL_{Cc}$*(R52A)-flag*. Km^R Sac^S	This work
pMR3921	pMR3552 with $cdnL_{Cc}$*(P54A)-flag*. Km^R Sac^S	This work
pMR4052	pMR3552 with $cdnL_{Cc}$*(Y127A)-flag*. Km^R Sac^S	This work
pMR4151	pET15b construct to overexpress $cdnL_{Cc}$. Amp^R	This work
pMR4208	pMR3552 with $cdnL_{Cc}$*(W90A)-flag*. Km^R Sac^S	This work
pMR4209	pMR3552 with $cdnL_{Cc}$*(R92A/R93A)-flag*. Km^R Sac^S	This work
pMR4210	pMR3552 with $cdnL_{Cc}$*(R130A)-flag*. Km^R Sac^S	This work
pMR4292	pET15b construct to overexpress $cdnL_{Cc}$*(R92A/R93A)*. Amp^R	This work
pMR4293	pET15b construct to overexpress $cdnL_{Cc}$*(R130A)*. Amp^R	This work
pMR4389	pVGFPC-2 with *flag*-$cdnL_{Cc}$ under P_{van} control. Km^R	This work
pMR4390	pVGFPC-2 with $cdnL_{Cc}$-*flag* under P_{van} control. Km^R	This work
pMR4396	pVGFPC-2 with *flag*-$cdnL_{Cc}$*(DD)* under P_{van} control. Km^R	This work
pMR4412	pVGFPC-5 with $cdnL_{Cc}$*(R130A)-flag* under P_{van} control. Tc^R	This work
pMR4413	pMR3552 with $cdnL_{Cc}$*(DD)*. Km^R Sac^S	This work
pMR4442	pMR3552 with $cdnL_{Cc}$. Km^R Sac^S	This work
pMR4640	pMR3552 with $cdnL_{Cc}$*(P54A)*. Km^R Sac^S	This work
pMR4641	pMR3552 with $cdnL_{Cc}$*(R130A)*. Km^R Sac^S	This work
pNPTS138	Allele exchange vector. Km^R Sac^S	M.R. Alley (unpublished)

pNPTS138-Δ*cdnL*$_{Cc}$	pNPTS138 with genomic regions flanking *cdnL*$_{Cc}$ to generate a suicide plasmid to delete *cdnL*$_{Cc}$	This work
pNPTS138-Δ*cdnL*$_{Cc}$-Ω	Suicide plasmid to replace *cdnL*$_{Cc}$ with $Strep^R$/$Spec^R$ omega (Ω) cassette. Km^R	This work
pNPT228-*gcrA*P-*lacZ*-Ω	Plasmid used to obtain a Ω cassette	64
pRK290-*cdnL*$_{Cc}$-*UTR*	pJGZ290 with P_{cdnLCc}::*lacZ*	This work
pRXMCS-6	Low-copy vector for xylose-inducible expression. Cm^R	27
pRXMCS-6-*cdnL*$_{Cc}$	pRXMCS-6 with *cdnL*$_{Cc}$ under P_{xyl} control. Cm^R	This work
pUT18	Vector for N-terminal fusion constructs to the T18 fragment of CyaA for bacterial two-hybrid analysis. Amp^R	55
pUT18C	Vector for C-terminal fusion constructs to the T18 fragment of CyaA for bacterial two-hybrid analysis. Amp^R	55
pVGFPC-2	Integrative shuttle vector for expressing proteins under P_{van} control in *C. crescentus*. Km^R	27
pVGFPC-5	Integrative shuttle vector for expressing proteins under P_{van} control in *C. crescentus*. Tc^R	27
pXGFPC-2	Vector used to integrate P_{rrnA}::*lacZ* reporter at a heterologous chromosomal site Km^R	27

Km^R, Tc^R, Cm^R: resistance to kanamycin, tetracycline and chloramphenicol, respectively. Sac^S: sensitivity to sucrose.

Supplementary References

61. Ely, B. Genetics of *Caulobacter crescentus*. *Methods Enzymol.* **204**, 372-384 (1991).

62. Evinger, M. & Agabian, N. Envelope-associated nucleoid from *Caulobacter crescentus* stalked and swarmer cells. *J. Bacteriol.* **132**, 294-301 (1977).

63. Tsai, J. W. & Alley, M. R. Proteolysis of the *Caulobacter* McpA chemoreceptor is cell cycle regulated by a ClpX-dependent pathway. *J. Bacteriol.* **183**, 5001-5007 (2001).

64. Collier, J., Murray, S. R. & Shapiro, L. DnaA couples DNA replication and the expression of two cell cycle master regulators. *EMBO J.* **25**, 346-356 (2006).
